# Molecular Docking Reveals Ivermectin and Remdesivir as Potential Repurposed Drugs Against SARS-CoV-2

**DOI:** 10.3389/fmicb.2020.592908

**Published:** 2021-01-25

**Authors:** Ahmad F. Eweas, Amr A. Alhossary, Ahmed S. Abdel-Moneim

**Affiliations:** ^1^Department of Pharmaceutical and Medicinal Chemistry, National Research Centre, Cairo, Egypt; ^2^Department of Science, University of Technology and Applied Sciences Rustaq, Rustaq, Oman; ^3^Rehabilitation Research Institute of Singapore, Nanyang Technological University, Singapore, Singapore; ^4^Microbiology Department, Virology Division, College of Medicine, Taif University, Taif, Saudi Arabia

**Keywords:** antiviral, chloroquine/hydroxychloroquine, COVID-19, coronavirus disease, favipiravir, ivermectin, remdesivir (GS-5734), SARS-CoV-2

## Abstract

SARS-CoV-2 is a newly emerged coronavirus that causes a respiratory disease with variable severity and fatal consequences. It was first reported in Wuhan and subsequently caused a global pandemic. The viral spike protein binds with the ACE-2 cell surface receptor for entry, while TMPRSS2 triggers its membrane fusion. In addition, RNA dependent RNA polymerase (RdRp), 3′–5′ exoribonuclease (nsp14), viral proteases, N, and M proteins are important in different stages of viral replication. Accordingly, they are attractive targets for different antiviral therapeutic agents. Although many antiviral agents have been used in different clinical trials and included in different treatment protocols, the mode of action against SARS-CoV-2 is still not fully understood. Different potential repurposed drugs, including, chloroquine, hydroxychloroquine, ivermectin, remdesivir, and favipiravir, were screened in the present study. Molecular docking of these drugs with different SARS-CoV-2 target proteins, including spike and membrane proteins, RdRp, nucleoproteins, viral proteases, and nsp14, was performed. Moreover, the binding affinities of the human ACE-2 receptor and TMPRSS2 to the different drugs were evaluated. Molecular dynamics simulation and MM-PBSA calculation were also conducted. Ivermectin and remdesivir were found to be the most promising drugs. Our results suggest that both these drugs utilize different mechanisms at the entry and post-entry stages and could be considered potential inhibitors of SARS-CoV-2 replication.

## Introduction

SARS-CoV-2 emerged in 2019 as the causative agent of a pneumonia outbreak in Wuhan, Hubei Province, China (Zhu et al., 2020). The disease outbreak spread globally, causing a pandemic with dozens of millions laboratory-confirmed cases (WHO, 2020) with more folds of the infections could be passed undetected (Hosein et al., 2020). The disease has resulted in more than 1,500,000 deaths as of 12 December 2020 (WHO, 2020).

SARS-CoV-2 belongs to the subgenus *Sarbecovirus*, genus *Betacoronavirus* and family *Coronaviridae*. The virus uses the angiotensin-converting enzyme 2 (ACE-2) cell receptor to enter cells (Hoffmann et al., 2020). The SARS-CoV-2 genome consists of ∼29.8 kb nucleotides; it possesses 14 open reading frames (ORFs) encoding 27 proteins (Wu A. et al., 2020). The 5′ two-thirds of the viral genome encodes the replicase gene. It contains two ORFs: ORF1a and ORF1b. ORF1a/b encodes two polyproteins by polymerase frameshifting; these are then post-translationally cleaved into 15 non-structural proteins (nsps): nsp1–10 and nsp12–16. The rest of the genome encodes four structural proteins [spike protein (S protein), envelope protein (E protein), membrane protein (M protein), and nucleocapsid protein (N protein)], in addition to eight accessory proteins (3a/3b, p6, 7a/7b, 8b, 9b, and ORF14) (Wu A. et al., 2020).

The S protein is proteolytically cleaved by type-II transmembrane serine protease (TMPRSS2) into S1 and S2 subunits (Hoffmann et al., 2020). The former subunit binds to the host cell surface receptor, while the latter is responsible for the fusion of the viral envelope and the cell membrane. The M protein is one of the most abundant envelope proteins. It plays an important role in determining the morphology of the virus. The E protein is present in a small amount on the envelope; however, it is important for the assembly and release of the virus. The N protein binds to the viral genome and forms the nucleocapsid of the virus (Abdel-Moneim and Abdelwhab, 2020). The replicase proteins encode the papain-like protease (PLpro) and the serine-type protease or main protease (Mpro) (Ziebuhr et al., 2000; Mielech et al., 2014). In addition, many other nsps, including RNA-dependent RNA polymerase (RdRp; nsp12) (Xu et al., 2003), RNA helicase (nsp13) (Ivanov et al., 2004), N7 MTase and 3′–5′ exoribonuclease (nsp14) (Eckerle et al., 2010), form the replicase–transcriptase complex (RTC), which is essential for RNA replication and transcription. The accessory proteins are also involved in viral replication and pathogenesis (Zhao et al., 2012; Fehr and Perlman, 2015).

Although extensive research has been conducted on SARS-CoV-2, no approved drug is available against the virus so far. Hence, there is an urgent need to develop sensitive and effective antiviral agents against zoonotic coronaviruses, including SARS-CoV-2. The registration of new antiviral drugs takes a long time; therefore, many studies have been conducted to screen the efficacy of already approved drugs against SARS-CoV-2. Many drugs have been found to possess potential antiviral activity against SARS-CoV-2. These include old antimalarial drugs (chloroquine phosphate, chloroquine, and hydroxychloroquine) (Wang M. et al., 2020), an anthelmintic drug (ivermectin) (Caly et al., 2020), viral RNA polymerase inhibitors (remdesivir and favipiravir) (National Health Commission of the People’s Republic of China, 2020; Wang M. et al., 2020) and viral protease inhibitors (Mugisha et al., 2020). Although some of such drugs possess know antiviral action (Gurung, 2020; Shannon et al., 2020; Wang M. et al., 2020; Yin et al., 2020), however, their possible antiviral effects on different viral proteins are not fully understood. Therefore, the present study aimed to perform molecular docking in order to assess the binding affinity of different viral proteins to different drugs with potential antiviral activities against SARS-CoV-2.

## Methodology

### Protein Retrieval and Preparation

The 3D structures of recently identified SARS-CoV-2 proteins, namely the S glycoprotein (PDB ID = 6VXX) (Walls et al., 2020), RdRp (PDB ID = 67M1) (Gao et al., 2020), Mpro (PDB ID = 6Y2E) (Zhang et al., 2020), PLpro (PDB ID = 6W9C) (Osipiuk et al., 2020), and the N protein (PDB ID = 6VYO) (Kang et al., 2020), were obtained from the RCSB Protein Data Bank^1^. On the other hand, the 3D structure of the viral M protein was not available; therefore, structural protein sequences of SARS-CoV-2 were downloaded from the NCBI Protein database (Accession No. QJA17755). Homology modeling of the viral proteins was performed using the SWISS-MODEL server^2^ with default settings. The M protein sequence of SARS-CoV-2 was entered in FASTA format, and the 3D homology model was retrieved from the SWISS-MODEL server as a PDB file and used for the docking process. Similarly, because of the lack of experimental 3D structure of the non-structural protein Nsp14 the sequence of SARS-CoV-2 ExoN/nsp14 (P0DTD1) was used to build a 3D homology model in the SWISS-MODEL web server based on the x-ray structure of Nsp14 from SARS-CoV (PDB ID: 5C8S, chain B) (Gurung, 2020).

The 3D structure of human ACE-2 was downloaded from the RCSB Protein Data Bank (see text footnote 1) (PDB ID = 1R42). However, 3D x-ray crystallographic data of TMPRSS2 were not available; therefore, the sequence of human TMPRSS2 (O15393) was retrieved from UniProt (UniProt Consortium, 2019), and loaded into the SWISS-MODEL server (see text footnote 2) with default settings to create three different 3D homology models of the protein. The top-ranked homology model built using the serine protease hepsin as the template (PDB ID = 5CE1) was subjected to protein preparation and optimization using the default protein preparation protocol in the Molegro Virtual Docker (MVD) software. Finally, the verified homology model of TMPRSS2 with good quality was used for molecular docking studies.

### Ligand Preparation

The 2D structures of all the drugs used in the study (chloroquine, hydroxychloroquine, ivermectin, remdesivir, favipiravir, lopinavir, and camostat), human ACE-2 cell receptor and cellular serine protease TMPRSS2 were compiled using ChemDraw, and the 3D structures of all the drug ligands were constructed using Chem3D ultra 15.0 software (Molecular Modeling and Analysis; Cambridge Soft Corporation, United States 2014). Then, the constructed structures were energetically minimized using MOPAC (semi-empirical quantum mechanics) (Job Type with 100 iterations and minimum RMS gradient of 0.01) and saved as an MDL Molfile (^∗^.mol).

### Molecular Docking

The 3D protein structures of all the proteins being studied were loaded onto the MVD 6.0 (2013) platform for the docking process. Potential binding sites (referred to as cavities) were identified using the built-in cavity detection algorithm of MVD. For each PDB file, protein preparation was performed using the default parameters in MVD before conducting the docking experiment. Subsequently, the docking process between different ligands and active sites of different protein structures was performed using the MolDock score as the scoring function of MVD with a grid resolution of 0.30 Å. The number of runs for each docking process was 10. Moreover, the maximum iterations were 2000, with an energy threshold of 100 Kcal/mol. The best conformations for each docking process were selected on the basis of the lowest docked binding energy.

### MD Simulation and MM-PBSA Calculation

To validate the accuracy of the ivermectin and remdesivir results, a short molecular dynamics (MD) simulation for each of the predicted receptor-ligand complexes was conducted. All simulations were conducted using GROMACS 2018 (Abraham et al., 2015), employing AMBER 99SB-ILDN force field (Maier et al., 2015). For every ligand-receptor complex, all receptor non-terminal missing loops/atoms were modeled using MODELLER (Sali and Blundell, 1993). Then missing hydrogen atoms were added using chimera (Pettersen et al., 2004) built-in method, putting special consideration on histidine residues to add their hydrogen atoms based on each histidine microenvironment. The complex was placed in an isotonic box (Main Protease and M-Protein were placed in a triclinic box, while the rest were placed in a dodecahedron cell). The box contains a neutralizing number of Na^+^ and Cl^–^ ions according to the protein charge. Topology parameters for the ligands were built using ACPYPE (Sousa da Silva and Vranken, 2012) and ANTECHAMBER (Wang et al., 2006) to generate general amber force field (GAFF) parameters. Then the regular steps of energy minimization, equilibration (NVT and NPT), then MD simulation with 2 femtosecond integration steps for 30 ns were conducted. The output trajectory was PBC corrected and the system was fitted to its start position based on the receptor’s backbone before further analysis was performed. The analysis included: (i) plotting the RMSD (root-mean-square deviation) of the ligand after fitting, (ii) plotting the number of hydrogen bonds between the ligand and the receptor, and (iii) MM-PBSA energy calculation. The first two measures were conducted using GROMACS suite, and the MM-PBSA energy was calculated using the g_MM-PBSA package (Baker et al., 2001; Kumari et al., 2014). For MM-PBSA calculation, the SAV model for calculation of the non-polar solvation energy was adopted, taking a sample every 1 ns. A ligand that could maintain its RMSD within 1 nm was considered fixed, less than 2 nm to be stable, but a ligand that possessed more than 2 nm RMSD to be non-stable.

## Results

### Binding Interactions of Selected Drugs With SARS-CoV-2 S Glycoprotein

**FIGURE 1:**
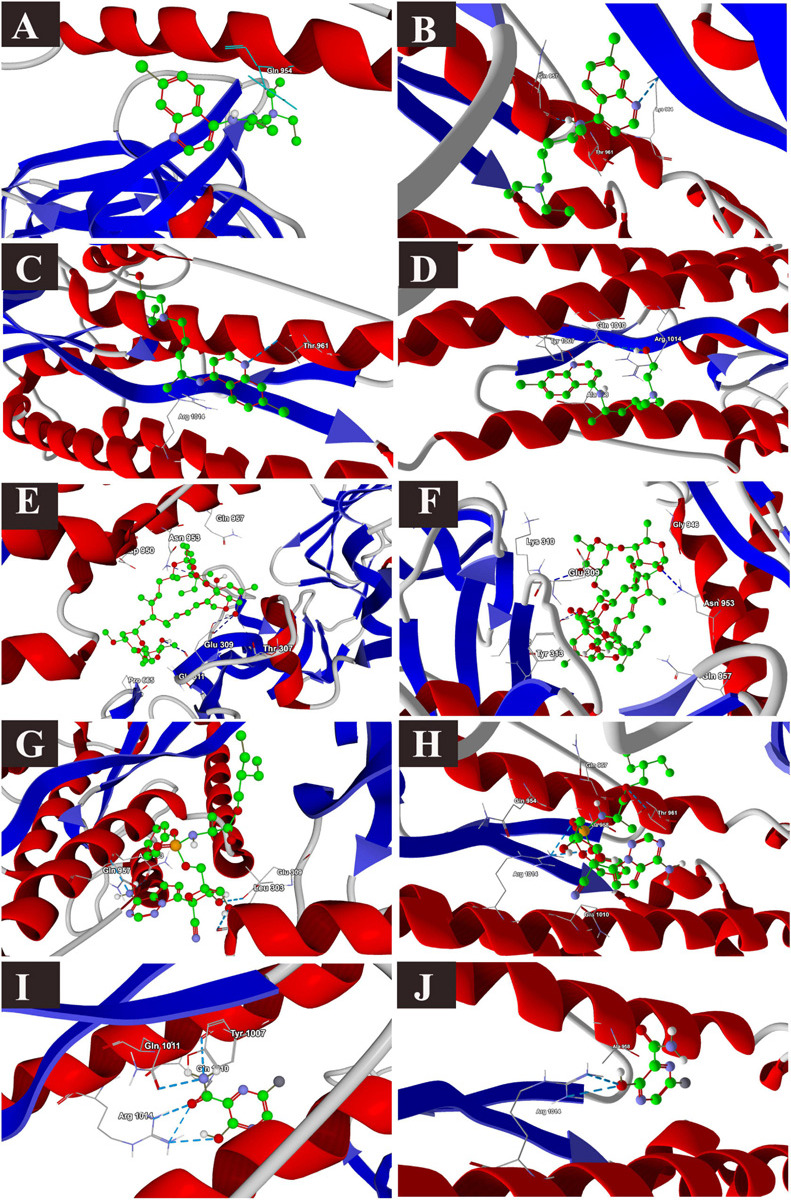
Binding interactions of selected drugs with SARS-CoV-2 spike glycoprotein. **(A)** chloroquine closed state, **(B)** chloroquine open state, **(C)** hydroxychloroquine closed state, **(D)** hydroxychloroquine, open state, **(E)** ivermectin closed state, **(F)** ivermectin, open state **(G)** remdesivir closed state, **(H)** remdesivir, open state, **(I)** favipiravir, closed state, **(J)** favipiravir, open state.

The binding energies of the selected drugs (Supplementary Figure 1) with the S glycoprotein (PDB ID = 6VXX) were studied. The docking results are given in terms of the MolDock score, interaction energy, H-bond energy and interacting amino acid residues present at the predicted active site of the protein (Table 1). Ivermectin showed the highest binding affinity to the predicted active site of the protein (MolDock score −140.584) and protein–ligand interactions (MolDock score −139.371). Moreover, it formed four H-bonds with Thr307, Glu309, Ile312, and Asn953 amino acid residues present at the predicted active site of the protein. In contrast, favipiravir showed the lowest binding affinity to the predicted active site of the protein (MolDock score −54.595) and protein–ligand interactions (MolDock score −68.539). Moreover, it formed one H-bond with the Arg1014 amino acid residue present at the predicted active site of the protein. Remdesivir showed considerable binding affinity (MolDock score −111.007) and protein–ligand interactions (MolDock score −122.699). It formed one H-bond with Leu303, Glu309, and Asn953. The 3D structural views of the ligand–binding site interactions are provided in Figure 1 and Table 1. By using open protein form, similar binding affinities were detected in all tested drugs, however, some variations in the interacting amino acids were detected (Figure 1 and Table 1).

**TABLE 1:** Docking of the SARS-CoV-2 spike protein (PDB id = 6VXX) to selected drugs.

**Drug**	**Protein form**	**MolDock score**	**Protein – ligand interactions**	**H-bonds**	**Interacting amino acids**
Chloroquine	Closed	–74.777	–93.770	0.000	—–
	Open	–72.019	–89.604	–4.967	Gln957, Lys964
Hydroxychloroquine	Closed	–84.427	–107.534	–4.970	Thr961
	Open	–89.421	–107.043	–4.329	Gln1010
Ivermectin	Closed	–140.584	–139.371	–9.530	Thr307, Glu309, Ile3,12, Asn953
	Open	–140.581	–127.699	–5.495	Lys310, Tyr313, Asn953
Remdesivir	Closed	–111.007	–122.699	–10.106	Leu303, Glu309, Asn953
	Open	–110.367	–111.887	–9.071	Thr961, Arg1014
Favipiravir	Closed	–59.437	–78.637	–12.229	Tyr1007, Gln1011, Arg1014
	Open	–54.176	–68.112	–6.432	Arg1014

*Docking parameters was conducted using Molegro Virtual Docker 6.0.*

### Binding Interactions of Selected Drugs With SARS-CoV-2 RdRp

**FIGURE 2:**
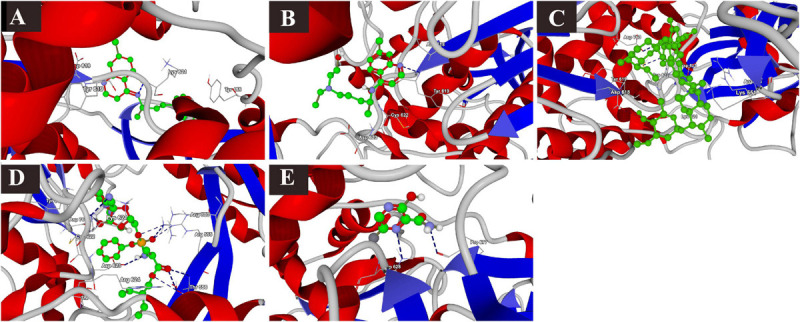
Binding interactions of selected drugs with SARS-CoV-2 RNA-dependent RNA polymerase. **(A)** chloroquine, **(B)** hydroxychloroquine, **(C)** ivermectin, **(D)** remdesivir, and **(E)** favipiravir.

The docking results of the selected drugs with SARS-CoV-2 RdRp (PDB ID = 6M71) are provided in Table 2. Remdesivir showed the highest binding affinity to the predicted active site of the protein (MolDock score −160.418) and protein–ligand interactions (MolDock score −173.270). Moreover, it formed seven H-bonds with Arg553, Arg555, Thr556, Tyr619, Lys621, Cys622, and Asp623 amino acid residues present at the predicted active site of the protein. Although ivermectin showed considerable binding affinity to the predicted active site of the protein (MolDock score −149.9900) and protein–ligand interactions (MolDock score −147.608), it formed H-bonds with only two amino acids: Cys622 and Asp760. The 3D structural views of the ligand–binding site interactions are provided in Figure 2.

**TABLE 2:** Docking of the RdRp and nsp14 to selected drugs.

	**Viral protein**	**MolDock score**	**Protein – Ligand interactions**	**H-bonds**	**Interacting amino acids**
Chloroquine	RdRp	–90.087	–115.579	–2.500	Lys621
	nsp14	–101.345	–118.748	–0.556	Tyr368
Hydroxychloroquine	RdRp	–100.690	–108.835	–2.491	Tyr619
	Nsp14	–95.934	–115.677	–10.225	Gly333, ASP352, Trp385, Cys387
Ivermectin	RdRp	–149.990	–147.608	–13.551	Cys622, Asp760
	Nsp14	–212.265	–215.323	–13.079	Gln313, Asn334, Ala353, Tyr386, Cys387
Remdesivir	RdRp	–160.418	–173.270	–24.247	Arg553, Arg555, Thr556, Tyr619, Lys621, Cys622, Asp623
	Nsp14	–137.866	–165.993	–8.666	Trp385, Asn386, Cys387, Gln354
Favipiravir	RdRp	–63.807	–77.835	–4.999	Asn628, Pro677
	Nsp14	–49.083	–63.368	–5.969	Asp352, Ala353

*RdRp: RNA dependent RNA polymerase protein (PDB id = 6M71). Nsp14: The 3D homology model of the SARS-CoV-2 nsp14 was built using the SWISS-MODEL online tool.*

### Binding Interactions of Selected Drugs With SARS-CoV-2 Nsp14 Protein

**FIGURE 3:**
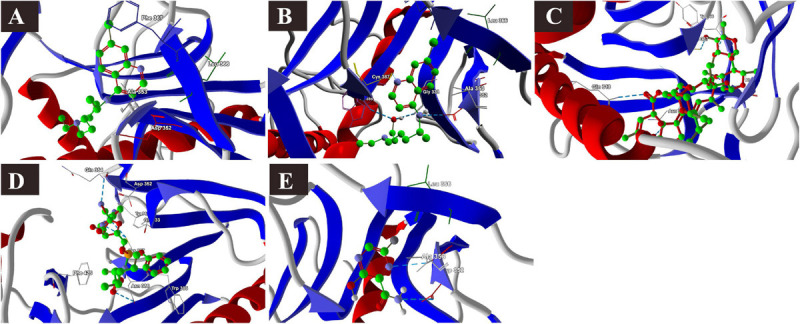
Binding interactions of selected drugs with SARS-CoV-2 NSP14. **(A)** chloroquine, **(B)** hydroxychloroquine, **(C)** ivermectin, **(D)** remdesivir, and **(E)** favipiravir.

The docking scores with nsp14 revealed that among the five tested drugs, ivermectin showed the highest binding affinity (MolDock score −212.265) and protein–ligand interactions (MolDock score −215.323). It formed five different H-bonds with the Gln313, Asn334, Ala353, Tyr386, and Cys387 amino acid residues present at the predicted active of the protein. Remdesivir showed relatively high binding affinities and protein–ligand interactions (Table 2). In contrast, favipiravir showed the lowest binding affinity (MolDock score −49.083) and protein–ligand interactions (MolDock score −63.368). It formed two H-bonds with Asp352 and Ala353 amino acid residues present at the predicted active site of the protein. The 3D structural views of the ligand–binding site interactions are provided in Figure 3.

### Binding Interactions of Selected Drugs With SARS-CoV-2 Mpro

**FIGURE 4:**
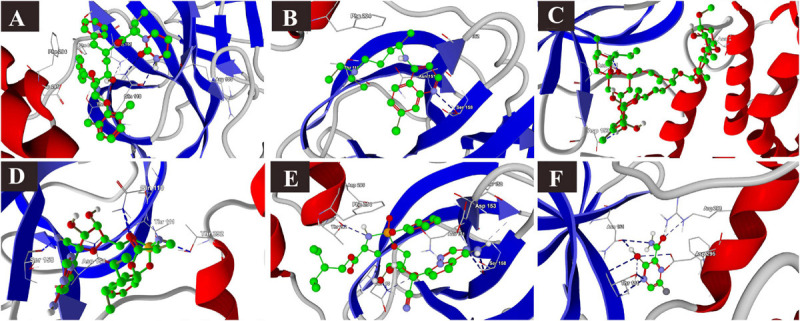
Binding interactions of selected drugs with SARS-CoV-2 main protease. **(A)** lopinavir, **(B)** chloroquine, **(C)** hydroxychloroquine, **(D)** ivermectin, **(E)** remdesivir, and **(F)** favipiravir.

Lopinavir, a protease inhibitor drug, was used as a reference drug. It showed the highest binding affinity (MolDock score −114.628) and protein–ligand interactions (MolDock score −159.244) and formed three H-bonds with Gln110, Thr111, and Asn151 amino acid residues present at the predicted active site of the protein. Among the tested drugs, remdesivir showed the highest binding affinity (MolDock score −123.033) and protein–ligand interactions (MolDock score −137.126). It formed five H-bonds with Gln110, Thr11, Asn151, Ser158, and Thr292 amino acid residues present at the predicted active site of the protein. This was followed by ivermectin, which also showed high binding affinity (MolDock score −114.860) and protein–ligand interactions (MolDock score −126.234). It formed three H-bonds with Asn151, Asp153, and Asn2033 amino acid residues present at the predicted active site of the protein. Hydroxychloroquine, showed high binding affinity (MolDock score −95.414) and protein–ligand interactions (MolDock score −112.507). It formed three H-bonds with Gln109, Ser158, and Asn203 amino acid residues present at the predicted active site of the protein. Meanwhile, chloroquine also showed high binding affinity (MolDock score −93.634) and protein–ligand interactions (MolDock score −107.868), however, formed two H-bonds only with Ser158. In contrast, favipiravir did not show considerable binding to Mpro (Table 3 and Figure 4).

**TABLE 3:** Docking of SARS-CoV-2 Mpro and PLpro against different drugs.

**Drug**	**Viral proteins**	**MolDock score**	**Protein – ligand interactions**	**H-bonds**	**Interacting amino acids**
Lopinavir	Mpro	–114.628	–159.244	–10.981	Gln110, Thr111, Asn151
	PLpro	–123.992	–149.066	–2.542	Asn156, Try171
Chloroquine	Mpro	–93.634	–107.868	–2.500	Ser158
	PLpro	–84.358	–87.406	–7.268	Thr74, Arg82, Asn156
Hydroxychloroquine	Mpro	–95.414	–112.507	–5.438	Gln109, Ser158, Asn203,
	PLpro	–90.097	–89.041	–2.500	Tyr154
Ivermectin	Mpro	–114.860	–126.234	–7.944	Asn151, Asp153, Asn203
	PLpro	–180.765	–165.337	–21.212	Thr74, Asn128, Gln174, Leu178
Remdesivir	Mpro	–123.033	–137.126	–20.588	Gln110, Thr111, Asn151, Ser158, Thr292
	PLpro	–141.949	–137.610	–20.198	Thr74, Thr75, Asp76, Ala153, His175
Favipiravir	Mpro	–58.123	–50.902	–19.210	Thr111, Asn151, Arg298
	PLpro	–58.161	–72.140	–13.428	Phe173, Gln174, Leu178, Val202

*Mpro, Main protease (PDB id = 6Y2E); PLpro, Papain like protease (PBD id: 6W9C).*

### Binding Interactions of Selected Drugs With SARS-CoV-2 PLpro

**FIGURE 5:**
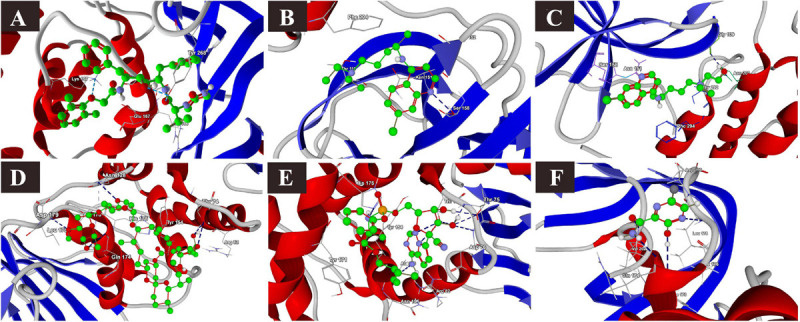
Binding interactions of selected drugs with SARS-CoV-2 papain-like protease. **(A)** lopinavir, **(B)** chloroquine, **(C)** hydroxychloroquine, **(D)** ivermectin, **(E)** remdesivir, and **(F)** favipiravir.

The docking results of the selected drugs with SARS-CoV-2 PLpro (PDB ID = 6W9C) are provided in Table 3. Ivermectin and remdesivir bound efficiently to PLpro. Ivermectin showed the highest binding affinity to the predicted active site of the protein (MolDock score −180.765) and protein–ligand interactions (MolDock score −165.337). Moreover, it formed four H-bonds with Thr74, Asn128, Gln174, and Leu178 amino acid residues present at the predicted active site of the protein. Similarly, remdesivir showed high binding affinity (MolDock score −141.949) and protein–ligand interactions (MolDock score −137.610). It formed six H-bonds with Thr74, Thr75, Asp76, Ala153, and His175 amino acid residues present at the predicted active site of the protein. The 3D structural visualizations of the ligand–binding site interactions are provided in Figure 5.

### Binding Interactions of Selected Drugs With SARS-CoV-2 M Protein

**FIGURE 6:**
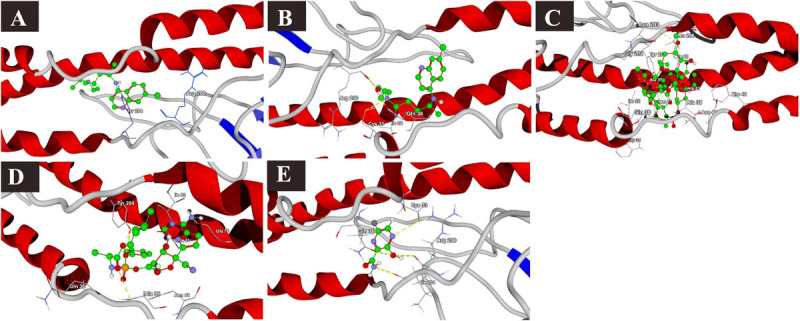
Binding interactions of selected drugs with SARS-CoV-2 M protein. **(A)** chloroquine, **(B)** hydroxychloroquine, **(C)** ivermectin, **(D)** remdesivir, and **(E)** favipiravir.

The binding affinities of the selected drugs to the M protein were studied. The docking results are provided in terms of the MolDock score, interaction energy, H-bond energy, and interacting amino acid residues present at the predicted active site of the protein (Table 4). The docking scores revealed that among the five drugs used for docking, remdesivir showed the highest binding affinity (MolDock score −180) and protein–ligand interactions (MolDock score −184.1560). Moreover, it formed one H-bond with the Tyr204 amino acid residue present at the predicted active site of the protein. Both hydroxychloroquine and ivermectin showed relatively high binding affinities and protein–ligand interactions (Table 4). In contrast, favipiravir showed the lowest binding affinity (MolDock score −59.714) and protein–ligand interactions (MolDock score −74.258). It formed three H-bonds with Cys33, Arg200, and Ile201 amino acid residues present at the predicted active site of the protein. The 3D structural views of the ligand–binding site interactions are provided in Figure 6.

**TABLE 4:** Docking of SARS-CoV-2 M and N proteins against different drugs.

**Drug**	**Viral protein**	**MolDock score**	**Protein – ligand interactions**	**H-bonds**	**Interacting amino acids**
Chloroquine	M	–111.217	–125.281	0.000	–
	N	–103.199	–123.884	–5.000	Tyr123, Trp132
Hydroxychloroquine	M	–124.335	–135.866	–7.441	Cyc33, Gln36
	N	–103.213	–120.328	–10.573	Arg68, Tyr123, Trp132
Ivermectin	M	–135.950	–153.929	–3.158	Trp31, Ala 81
	N	–136.473	–134.935	–10.777	Gln160, Leu161, Gly164, Thr166
Remdesivir	M	–180.716	–184.156	–8.744	Tyr204
	N	–126.986	–151.502	–21.271	Asn75, Asn77, His145, Asn154
Favipiravir	M	–59.714	–74.258	–7.998	Cys33, Arg200, Ile201
	N	–71.807	–93.349	–21.578	Phe66, Pro67, Gly69, Gln70, Tyr123, Trp132, Ala134

*M, Matrix protein; N, Nucleocapsid phosphoprotein (PDB id: 6VYO).*

### Binding Interactions of Selected Drugs With SARS-CoV-2 N Phosphoprotein

**FIGURE 7:**
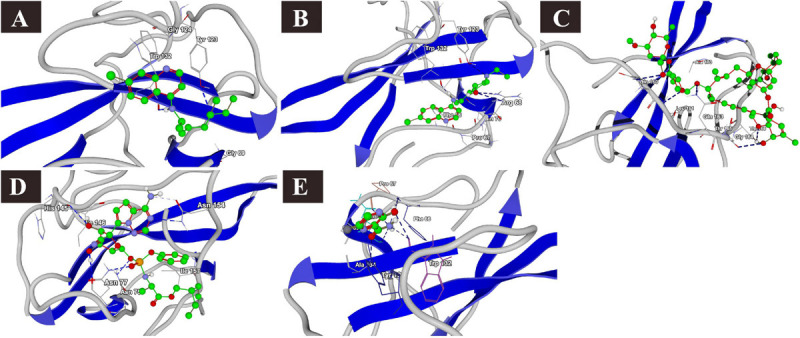
Binding interactions of selected drugs with SARS-CoV-2 nucleocapsid phosphoprotein. **(A)** chloroquine, **(B)** hydroxychloroquine, **(C)** ivermectin, **(D)** remdesivir, and **(E)** favipiravir.

The docking results of the selected drugs with the SARS-CoV-2 N phosphoprotein (PDB ID = 6VYO) are provided in Table 4. Ivermectin showed the highest binding affinity to the predicted active site of the protein (MolDock score −136.473) and high protein–ligand interactions (MolDock score −134.935). Moreover, it formed four H-bonds with Gln160, Leu161, Gly164, and Thr166 amino acid residues present at the predicted active site of the protein. In addition, remdesivir showed high binding affinity (MolDock score −126.986) and higher protein–ligand interactions (MolDock score −151.502). It formed hydrogen bonds with Asn75, Asn77, His145, and Asn154 (Figure 7).

### Binding Interactions of Selected Drugs With Human ACE-2 Protein

MLN-4760, an ACE-2 inhibitor, was used as a positive control in the docking process. The docking results (Table 5) revealed that ivermectin showed the highest binding affinity to the active site of the protein (MolDock score −159.754) and protein–ligand interactions (MolDock score −176.371). Moreover, it formed five H-bonds with Arg273, Glu398, Ser511, Arg514, and Tyr515 amino acid residues present at the predicted active site of the protein. This was followed by remdesivir, which showed high binding affinity (MolDock score −147.110) and protein–ligand interactions (MolDock score −155.467). It formed four H-bonds with Asn394, Glu402, Glu406, and Arg514 amino acid residues present at the predicted active site of the protein. Hydroxychloroquine but not chloroquine bound to the human ACE-2 receptor with considerable binding force (MolDock score −120.165) and protein–ligand interactions (MolDock score −134.164). Moreover, it formed H-bonds with Arg273, Ala348, Glu375, and Arg514 amino acid residues. In comparison, the reference ligand MLN-4760 showed high binding affinity (MolDock score −154.736) and protein–ligand interactions (MolDock score −136.555). It formed five H-bonds with Arg273, His345, Pro346, Thr37, and Tyr515 amino acid residues present at the predicted active site of the protein (Supplementary Figure 2).

**TABLE 5:** Docking scores of different drugs against human ACE-2 protein.

**Drug**	**MolDock score**	**Protein – ligand interactions**	**H-bonds**	**Interacting amino acids (H-bonding)**
MLN-4760	–154.736	–136.555	–15.331	Arg273, His345, Pro346, Thr371, Tyr515
Chloroquine	–101.590	–121.396	–4.752	Asn394, Gly395
Hydroxychloroquine	–120.165	–134.164	–12.276	Arg273, Ala348, Glu375, Arg514
Ivermectin	–159.754	–176.371	–22.162	Arg273, Glu398, Ser511, Arg514, Tyr515
Remdesivir	–147.110	–155.467	–17.407	Asn394, Glu402, Glu406, Arg514
Favipiravir	–68.998	–83.943	–14.432	Asn394, Gly395, Asn397, Glu398, Arg514

### Binding Interactions of Selected Drugs With Human TMPRSS2 Protein

Camostat, a known TMPRSS2 inhibitor, was used as a reference ligand for the docking study of the selected drugs with the predicted active site of the TMPRSS2 homology model. The docking results revealed that ivermectin showed the highest binding affinity to the active site of the protein (MolDock score −174.971) and protein–ligand interactions (MolDock score −180.548). Moreover, it formed five H-bonds with Cys297, Glu299, Gln438, Gly462, and Gly464 amino acid residues present at the predicted active site of the protein. On the other hand, the reference drug camostat showed a binding energy of −131.596 (MolDock score), showed protein–ligand interactions (MolDock score −163.157) and formed five H-bonds with Glu299, Gln438, Asp435, Ser436, and Val473 amino acid residues present at the predicted active site of the protein (Table 6 and Supplementary Figure 2).

**TABLE 6:** Docking scores and functions of drugs against human TMPRSS2 protein.

**Drug**	**MolDock score**	**Protein – ligand interactions**	**H-bonds**	**Interacting amino acids**
Camostat (+ control)	–131.596	–163.157	–15.210	Gln438, Asp435, Ser436, Val473
Chloroquine	–92.252	–116.167	–0.908	Ser436
Hydroxychloroquine	–113.593	–139.090	–8.426	His 296, Ser441, Ser436
Ivermectin	–174.971	–180.548	–7.493	Glu299, Gln438, Ser441
Remdesivir	–117.540	–142.941	–6.311	Ser441, Gln438
Favipiravir	–84.160	–98.110	–18.231	Gly265, Trp267, Gln270, Leu360, Ile314

### RMSD and MM-PBSA Energy

**FIGURE 8:**
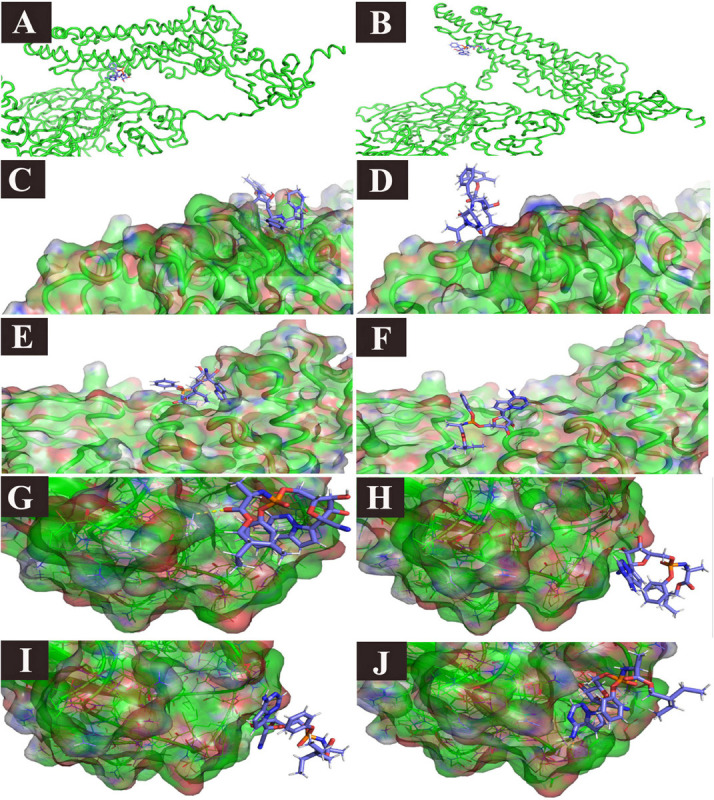
Ligand/protein complex MD simulation: selected examples. **(A)** Remdesivir/spike (*t* = 0): spike closes, **(B)** Remdesivir/spike (*t* = 13 ns), the spike opens, and the ligand is still in place, **(C)** Lopinavir/PLpro (*t* = 0), the ligand is in place, **(D)** Lopinavir/PLpro (*t* = 26 ns), the ligand left the pocket. **(E)** Remdesivir/PLpro (*t* = 0), the ligand is in place, **(F)** Remdesivir/PLpro (*t* = 23 ns), the ligand moved to the other side of the pocket, **(G)** Remdesivir/NP (*t* = 2 ns), the ligand is in the pocket, **(H)** Remdesivir/NP (*t* = 21 ns), the pocket is obliterated, and the ligand left the pocket, but it is still attached to the receptor with two hydrogen bonds, and one Pi stacking, **(I)** Remdesivir/NP (*t* = 22 ns), the pocket is still obliterated, and the ligand left the pocket, but it is still attached to the receptor with one hydrogen bond. **(J)** Remdesivir/NP (*t* = 30), The pocket is open again and the ligand is restored in place.

All the studied receptor – ligand complexes showed ligand heavy atoms RMSD within the stable range, except RdRp/ivermectin, spike/remdesivir, NP/remdesivir, and PLpro with either lopinavir or remdesivir (Supplementary Figure 3). The free binding energy of the spike protein (open) was higher in ivermectin (−398.536 kJ/mol) than remdesivir (−232.973 kJ/mol). In the remdesivir/spike structure complex, although the simulation showed high RMSD value of the ligand heavy atoms, this high value is related to the major steric changes the protein underwent. The ligand managed to follow the change, keeping a stable number of two hydrogen bonds in the second half of the trajectory (Table 7 and Supplementary Figure 3 and Figures 8A,B). Although there was no significant difference between free binding energy for SARS-CoV-2 RdRp with ivermectin (−179.472 kJ/mol) and remdesivir (−345.437 kJ/mol), the ivermectin/RdRp complex was not stable (Supplementary Figure 3). The free binding energy for ivermectin with ExoN/NSP14 (−418.894 kJ/mol), was higher than that with remdesivir (−362.872 kJ/mol). The free binding energy of lopinavir/Mpro complex was −324.160 kJ/mol while lopinavir/PLpro complex showed low free binding energy (−134.961 kJ/mol). The lopinavir/PLpro complex was not successful since lopinavir left the pocket totally (Figures 8C,D). Similarly, ivermectin showed a higher free binding energy (−345.675 kJ/mol) with Mpro, and lower binding energy of −221.257 with PLpro. In contrast, remdesivir showed similar free binding energy for Mpro and PLpro; −222.664 kJ/mol and −209.861 kJ/mol, respectively. However, in the remdesivir/PLpro complex, the remdesivir moved from one side of the pocket to the other side (Figures 8E,F). The free binding energy of M protein with ivermectin was the highest among all the complexes (−516.656 kJ/mol) and it also showed higher free binding energy with NP was −334.190 than remdesivir (Table 7 and Supplementary Figure 2). Remdesivir free binding energy with NP protein (−169.744 kJ/mol) was lower than that detected with ivermectin (Table 7 and Supplementary Figure 2). The remdesivir was stabilized in the NP binding pocket in the beginning by 1–4 simultaneous hydrogen bonds, in addition to the hydrophobic attraction (force). Then, during the period *t* = 21 till *t* = 30, the pocket was obliterated. However, the ligand was kept near to its pocket, using 1–3 hydrogen bonds, until the pocket was opened. The ligand was then restored to its original position (Table 7 and Figures 8G–J). The free binding energy of human ACE-2 with ivermectin and remdesivir were −227.529 and −209.396, respectively. The remdesivir/human ACE-2 complex, just after the initial part of the simulation, showed a tendency to stabilize in place with 4–5 hydrogen bonds (Table 7 and Supplementary Figure 3). On the other hand, the free binding energy of human TMPRSS2/camostat complex was −266.882. High free binding energy of TMPRSS2/was also recorded with both ivermectin, and remdesivir, −293.245 and −249.480, respectively (Table 7).

**TABLE 7:** Calculated MM-PBSA energy term.

		**ΔE_*vdw*_**	**ΔE_*ele*_**	**ΔG_*PB*_**	**ΔE_*SAV*_**	**ΔE_*bind*_**
Spike (open)	Ivermectin	−288.129 ± 4.033	−41.310 ± 5.135	164.281 ± 4.602	−234.189 ± 4.359	−398.536 ± 7.050
	Remdesivir	−171.736 ± 9.300	−76.439 ± 10.074	153.940 ± 11.037	−139.485 ± 9.879	−232.973 ± 16.230
RdRp	Ivermectin	−126.760 ± 5.600	−92.355 ± 9.745	139.100 ± 11.769	−98.697 ± 7.709	−179.472 ± 11.756
	Remdesivir	−193.101 ± 3.364	−408.114 ± 16.285	418.823 ± 13.286	−162.517 ± 5.255	−345.437 ± 10.650
ExoN/NSP14	Ivermectin	−340.539 ± 4.512	−67.396 ± 4.426	257.748 ± 5.781	−268.284 ± 5.106	−418.894 ± 7.720
	Remdesivir	−296.809 ± 4.797	−115.109 ± 5.253	266.288 ± 4.943	−217.653 ± 4.513	−362.872 ± 8.280
Mpro	Lopinavir	−206.370 ± 6.746	−117.454 ± 6.121	161.074 ± 5.131	−161.033 ± 6.322	−324.160 ± 12.229
	Ivermectin	−250.618 ± 4.104	−68.502 ± 7.669	184.288 ± 6.553	−210.202 ± 4.445	−345.675 ± 8.403
	Remdesivir	−152.746 ± 3.849	−113.799 ± 10.644	173.207 ± 11.392	−129.400 ± 6.131	−222.664 ± 6.560
PLpro	Lopinavir	−85.353 ± 5.964	−61.714 ± 5.776	80.340 ± 4.292	−68.364 ± 6.112	−134.961 ± 8.836
	Ivermectin	−146.486 ± 3.737	−62.667 ± 4.795	107.319 ± 5.284	−119.582 ± 4.243	−221.257 ± 7.352
	Remdesivir	−138.183 ± 5.329	−109.431 ± 9.318	146.084 ± 6.842	−108.474 ± 5.077	−209.861 ± 10.284
M-Protein	Ivermectin	−360.352 ± 7.596	−71.537 ± 5.954	194.848 ± 5.027	−279.701 ± 5.213	−516.656 ± 11.960
	Remdesivir	−269.933 ± 3.138	−174.798 ± 6.584	255.597 ± 4.665	−203.809 ± 3.729	−392.521 ± 7.044
NP	Ivermectin	−191.202 ± 2.670	−161.261 ± 10.422	188.470 ± 6.860	−170.112 ± 4.216	−334.190 ± 7.554
	Remdesivir	−93.332 ± 4.791	−94.663 ± 9.078	101.078 ± 6.437	−82.833 ± 5.924	−169.744 ± 12.748
Human ACE-2	Ivermectin	−192.437 ± 2.894	−45.851 ± 5.840	155.668 ± 4.917	−144.682 ± 3.322	−227.529 ± 5.809
	Remdesivir	−163.363 ± 3.266	−73.073 ± 14.043	160.621 ± 9.253	−132.672 ± 4.823	−209.396 ± 10.720
Human TMPRSS2	CAMOSTAT	−159.207 ± 3.910	−188.365 ± 7.639	210.987 ± 5.771	−130.522 ± 4.341	−266.882 ± 8.643
	Ivermectin	−228.025 ± 5.972	−63.653 ± 8.620	175.498 ± 6.837	−176.753 ± 6.132	−293.245 ± 9.019
	Remdesivir	−208.849 ± 2.823	−113.873 ± 8.848	218.313 ± 5.047	−145.920 ± 4.196	−249.480 ± 8.422

*All numbers are given in kJ/mol. Δ*E_*vdW*_, van der Waals* interaction *energies*; Δ*E*_*ele*_, Coulombic energy; Δ*G_*PB*_*, Poisson–Boltzmann polar solvation energy; Δ*E*_*SAV*_, non-polar solvation energy; Δ*E_*bind*_*, free binding energy.*

## Discussion

Both ivermectin and remdesivir showed high binding affinity to different viral proteins and seem to be potential drugs against SARS-CoV-2; however, laboratory and clinical trials are needed, particularly for ivermectin. Both these drugs possess multidisciplinary actions.

Ivermectin showed high binding affinity to the viral S protein as well as the human cell surface receptors ACE-2 and TMPRSS2. In agreement to our findings, ivermectin was found to be docked between the viral spike and the ACE2 receptor (Lehrer and Rheinstein, 2020). The activation of the S protein by TMPRSS2 can make cathepsin L activity and low pH unnecessary for the viral envelope to fuse with the endosomal membrane (Glowacka et al., 2011). The molecular docking of ivermectin with TMPRSS2 suggested an important role of ivermectin in inhibiting the entry of the virus into the host cell, probably by increasing the endosomal pH. Moreover, it efficiently binds to both Mpro and binds also but to lesser extent to PLpro of SARS-CoV-2; therefore, it might also play a role in preventing the post-translational processing of viral polyproteins. The highly efficient binding of ivermectin to the viral N phosphoprotein and M protein is suggestive of its role in inhibiting viral replication and assembly. Accordingly, it may also be involved in inhibiting nuclear transport. In addition to the efficient binding of ivermectin to the N protein, previous studies have revealed that ivermectin inhibits IMPα/β1-mediated nuclear import of the N protein (Rowland et al., 2005; Timani et al., 2005; Wagstaff et al., 2012; Tay et al., 2013; Caly et al., 2020; Yang et al., 2020). A recent *in vitro* study revealed ∼5000-fold reduction of SARS-CoV-2 RNA at 48 h in Vero cells when ivermectin was added to the cells 2 h post-infection (Caly et al., 2020). The highly efficient binding of ivermectin to nsp14 confirms its role in inhibiting viral replication and assembly. It is well known that nsp14 is essential in transcription and replication. It acts as a proofreading exoribonuclease and plays a role in viral RNA capping by its methyl transferase activity (Ma et al., 2015).

Remdesivir showed high affinity to spike but formed unstable complex, however, it showed considerable high affinity to both TMPRSS2 and ACE-2 might denote its possible roles in blocking cellular receptor necessary for viral entry in addition of inhibiting TMPRSS2 induced membranes’ fusion required for the SARS-CoV-2 replication (Glowacka et al., 2011; Hoffmann et al., 2020). The affinity of remdesivir to bind human TMPRSS2 was also suggested in a previous study (Wu C. et al., 2020).

Remdesivir is an adenosine analog, it inhibits RNA strand elongation, thereby inhibiting viral replication (Warren et al., 2016). In addition, it showed high binding affinity to RdRp, as confirmed in the present study. A recent study found that remdesivir able to bind RdRp (Wu C. et al., 2020). In addition, SARS-CoV-2 RdRp in the apo form or in association with the RNA template primer can bind to remdesivir (Yin et al., 2020). The high binding affinity of remdesivir to both Mpro and PLpro of SARS-CoV-2 is suggestive of its potential as a protease inhibitor. Our assumption was confirmed by a recent *in silico* study (Deshpande et al., 2020), which reported that remdesivir efficiently binds to Mpro. In addition, remdesivir has been found to be more potent than lopinavir/ritonavir both *in vitro* and in MERS-CoV-infected mice (Sheahan et al., 2020). Remdesivir was found to have high affinity to SARS-CoV-2 main protease (Hall and Ji, 2020).

Remdesivir also showed high affinity to M and nsp14 proteins of SARS-CoV-2, further suggesting its role in inhibiting different steps of viral replication. M and S protein as well as M and N protein interactions are required for viral component assembly in host cells (Siu et al., 2008).

Although lopinavir was reported to show high affinity to SARS-CoV-2 main protease (Kumar et al., 2020), in the current study, it formed unstable complex which also agree with a previous study that found it unable to bind to Mpro and PLpro (Choy et al., 2020; Wu C. et al., 2020).

The binding affinities of chloroquine, hydroxychloroquine, and favipiravir were lower than those of ivermectin and remdesivir for all the tested proteins. However, chloroquine and hydroxychloroquine showed considerably high binding affinities to Mpro, the M protein, ACE-2, and TMPRSS2. Previous studies have revealed that hydroxychloroquine possesses wide-range antiviral activity (Savarino et al., 2006; Yan et al., 2013). In accordance with these findings, it was found that hydroxychloroquine exerts its antiviral effect against SARS-CoV by altering the pH and interfering with the glycosylation of the ACE-2 receptor (Vincent et al., 2005); this is considered to be the major antiviral action of hydroxychloroquine (Savarino et al., 2006). Interestingly, hydroxychloroquine interferes with glycosyltransferases (sugar-modifying enzymes) and inhibits quinone reductase 2 (Kwiek et al., 2004) and the biosynthesis of sialic acid (Vincent et al., 2005). In addition, it interferes with the fusion of the virus and the cellular endosome by altering the endosomal pH (Vincent et al., 2005). Although chloroquine phosphate has been found to successfully inhibit SARS-CoV-2 replication in different clinical trials (Wang M. et al., 2020; Yin et al., 2020), this drug did not show binding activity to the target proteins tested in the present study. Similarly, favipiravir is a purine analog that inhibits the elongation phase of RNA synthesis. It has shown promising effects in COVID-19 patients (Cai et al., 2020). Moreover, an *in vitro* study revealed that compared with chloroquine or remdesivir, only a high concentration of favipiravir (EC_50_*?* = *?*61.88 μM) could inhibit SARS-CoV-2 replication (Wang M. et al., 2020). However, in the present study, it did not show good binding affinity to any of the tested proteins.

Chloroquine, hydroxychloroquine, and favipiravir could exhibit antiviral effects through different proteins or intermediate protein–protein interactions; however, these were not screened in the present study.

In conclusion, both ivermectin and remdesivir could be considered potential drugs for the treatment of COVID-19. Ivermectin efficiently binds to the viral S protein as well as the human cell surface receptors ACE-2 and TMPRSS2; therefore, it might be involved in inhibiting the entry of the virus into the host cell. It also binds to Mpro and PLpro of SARS-CoV-2; therefore, it might play a role in preventing the post-translational processing of viral polyproteins. The highly efficient binding of ivermectin to the viral N phosphoprotein and nsp14 is suggestive of its role in inhibiting viral replication and assembly. Remdesivir may be involved in inhibiting post-entry mechanisms as it shows high binding affinity to N and M proteins, PLpro, Mpro, RdRp, and nsp14. Although the results of clinical trials for remdesivir are promising (Beigel et al., 2020; Wang Y. et al., 2020), similar clinical trials for ivermectin are recommended. Both these drugs exhibit multidisciplinary inhibitory effects at both viral entry and post-entry stages.

## Data Availability Statement

The original contributions presented in the study are included in the article/Supplementary Material, further inquiries can be directed to the corresponding author.

## Author Contributions

ASA-M contributed conception of the study and critically revised the manuscript. AFE conducted the molecular docking and wrote the first draft of the manuscript. AAA performed the molecular dynamics simulation. ASA-M, AFE, and AAA shared analyzing and discussing the results. All authors contributed to manuscript revision, read, and approved the submitted version.

## Conflict of Interest

The authors declare that the research was conducted in the absence of any commercial or financial relationships that could be construed as a potential conflict of interest.

## Footnotes

1 https://www.rcsb.org/

2 http://swissmodel.expasy.org/

## Supplementary Material

Supplementary Figure 1 Chemical structure of FDA drugs used in the present study.

Supplementary Figure 2 Binding interactions of selected drugs with human ACE-2 protein and TMPRSS2 protein. (1) Binding interactions of selected drugs with human ACE-2 protein. **(A)** MLN-476, **(B)** chloroquine, **(C)** hydroxychloroquine, **(D)** ivermectin, **(E)** remdesivir, and **(F)** favipiravir. (2) Binding interactions of selected drugs with human TMPRSS2 protein. **(A)** Camostat, **(B)** chloroquine, **(C)** hydroxychloroquine, **(D)** ivermectin, **(E)** remdesivir, and **(F)** favipiravir.

Supplementary Figure 3 RMSD and number of Hydrogen bonds. For every receptor, the RMSD and Hydrogen bonds number for each ligand were demonstrated.
