# Supplementary figures and images for: Molecular Docking Reveals Ivermectin and Remdesivir as Potential Repurposed Drugs Against SARS-CoV-2
Source: Front Microbiol. 2021 Jan 25;11:592908. doi: 10.3389/fmicb.2020.592908 (PMC7976659; doi:10.3389/fmicb.2020.592908)

**Suppl.1**

Chemical structure of FDA drugs used in the present study.


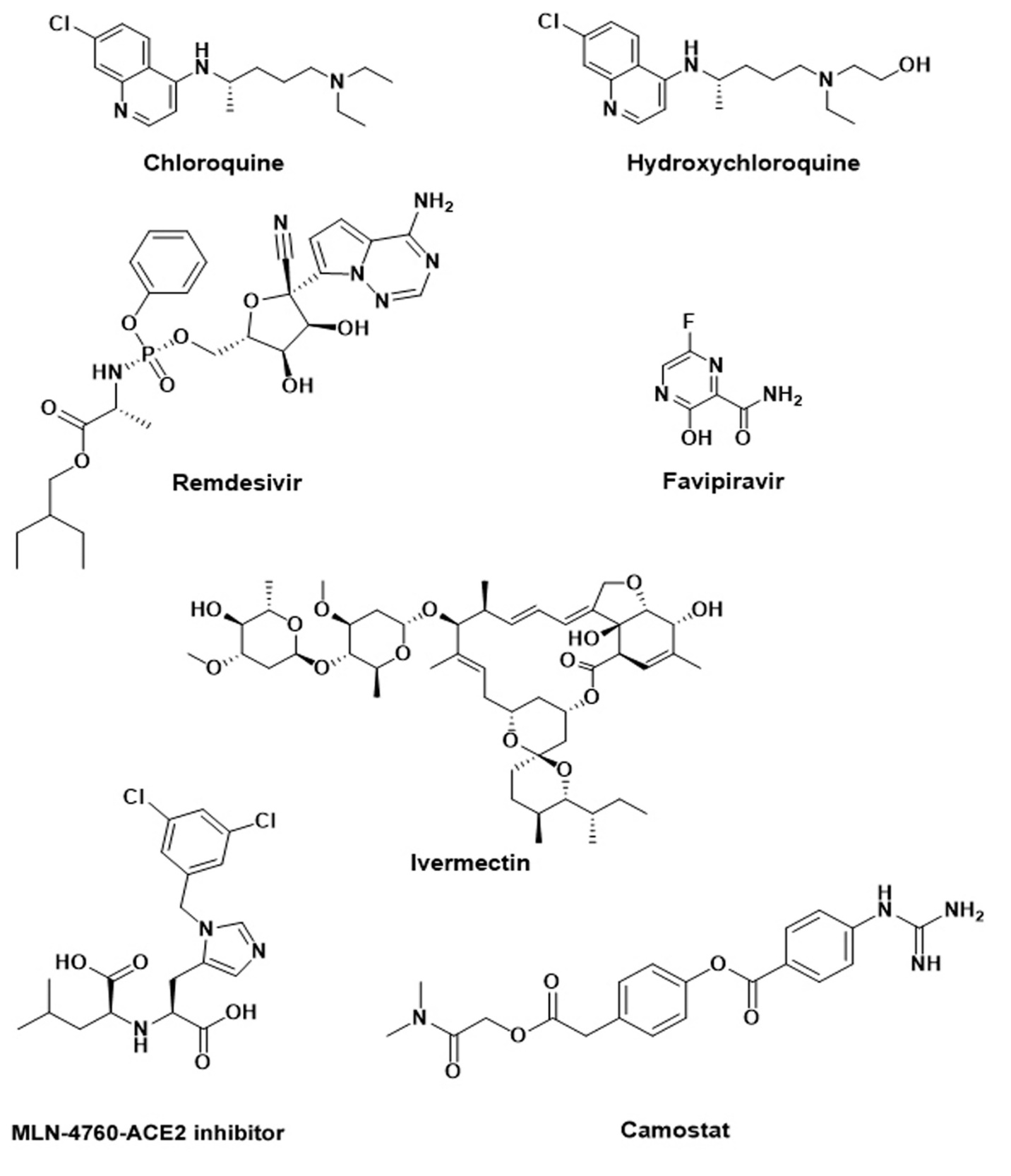

Supplement: Supplementary Figure 1 — Chemical structure of FDA drugs used in the present study. [file Data_Sheet_1.docx]
