# Supplementary material for: Molecular Docking Reveals Ivermectin and Remdesivir as Potential Repurposed Drugs Against SARS-CoV-2
Source: Front Microbiol. 2021 Jan 25;11:592908. doi: 10.3389/fmicb.2020.592908 (PMC7976659; doi:10.3389/fmicb.2020.592908)
Supplement: Supplementary Figure 2 — Binding interactions of selected drugs with human ACE-2 protein and TMPRSS2 protein. (1) Binding interactions of selected drugs with human ACE-2 protein. (A) MLN-476, (B) chloroquine, (C) hydroxychloroquine, (D) ivermectin, (E) remdesivir, and (F) favipiravir. (2) Binding interactions of selected drugs with human TMPRSS2 protein. (A) Camostat, (B) chloroquine, (C) hydroxychloroquine, (D) ivermectin, (E) remdesivir, and (F) favipiravir. [file Data_Sheet_2.docx]

**Suppl. 2**

**Binding interactions of selected drugs with human ACE-2 protein and TMPRSS2 protein**


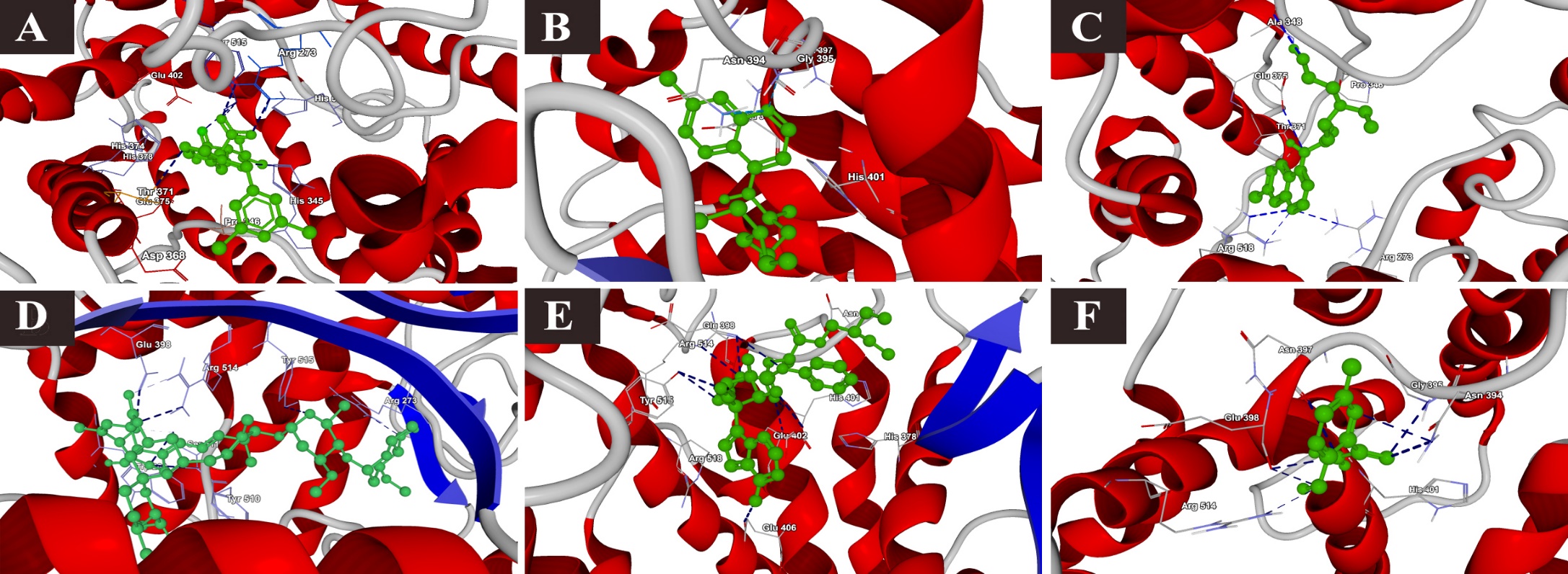
**1) Binding interactions of selected drugs with human ACE-2 protein**

a) MLN-476, b) chloroquine, c) hydroxychloroquine, d) ivermectin, e) remdesivir and f) favipiravir

**2) Binding interactions of selected drugs with human TMPRSS2 protein**


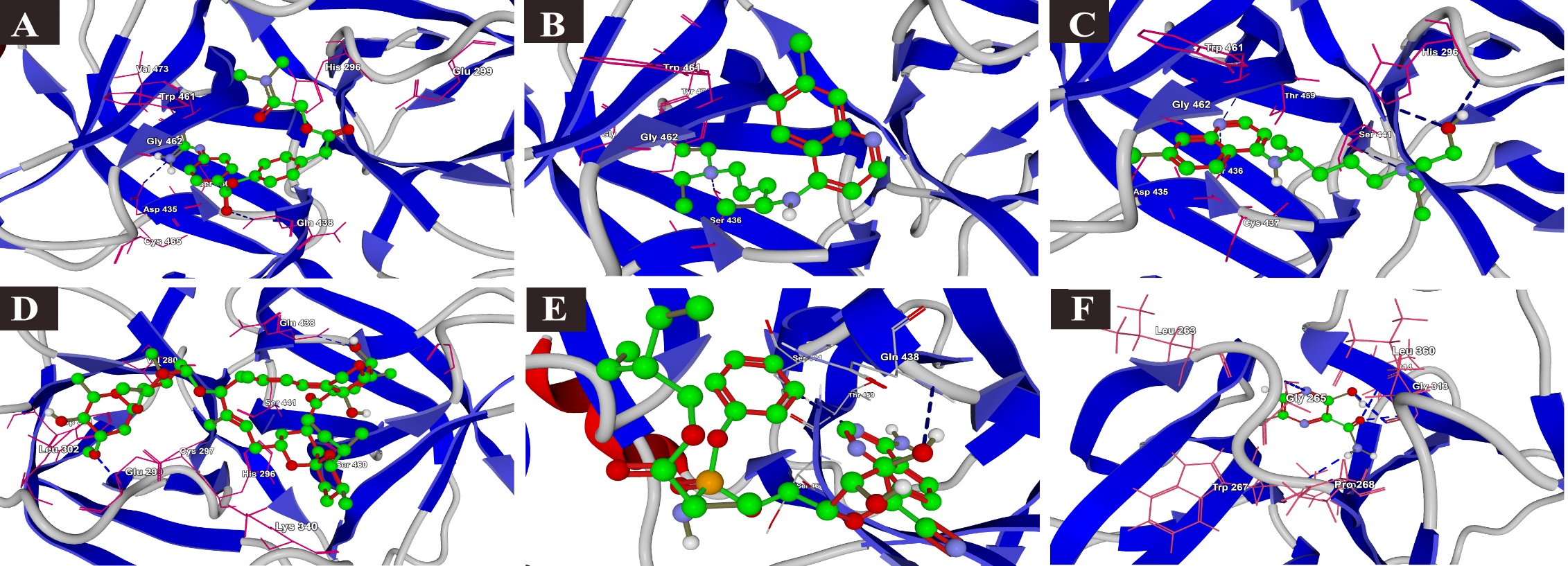


a) Comstat b) chloroquine, c) hydroxychloroquine, d) ivermectin, e) remdesivir and f) favipiravir.
