# Supplementary material for: Molecular Docking Reveals Ivermectin and Remdesivir as Potential Repurposed Drugs Against SARS-CoV-2
Source: Front Microbiol. 2021 Jan 25;11:592908. doi: 10.3389/fmicb.2020.592908 (PMC7976659; doi:10.3389/fmicb.2020.592908)
Supplement: Supplementary Figure 3 — RMSD and number of Hydrogen bonds. For every receptor, the RMSD and Hydrogen bonds number for each ligand were demonstrated. [file Data_Sheet_3.docx]

## Suppl. 3: RMSD and number of Hydrogen bonds.

For every receptor, the RMSD and Hydrogen bonds number for each ligand were demonstrated.

|  |  | **RMSD of ligand’s heavy atoms** | **Number of H bonds** |
| --- | --- | --- | --- |
| **Spike (open)** | **Ivermectin** | 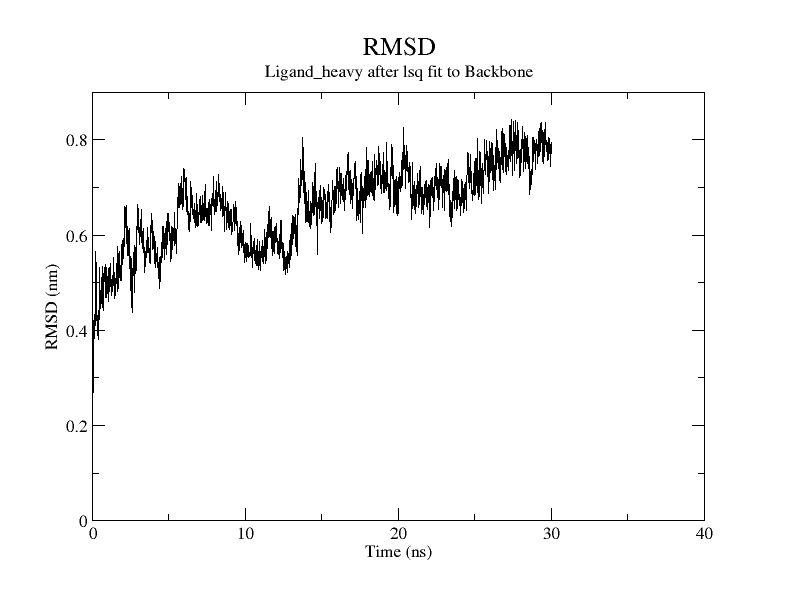 | 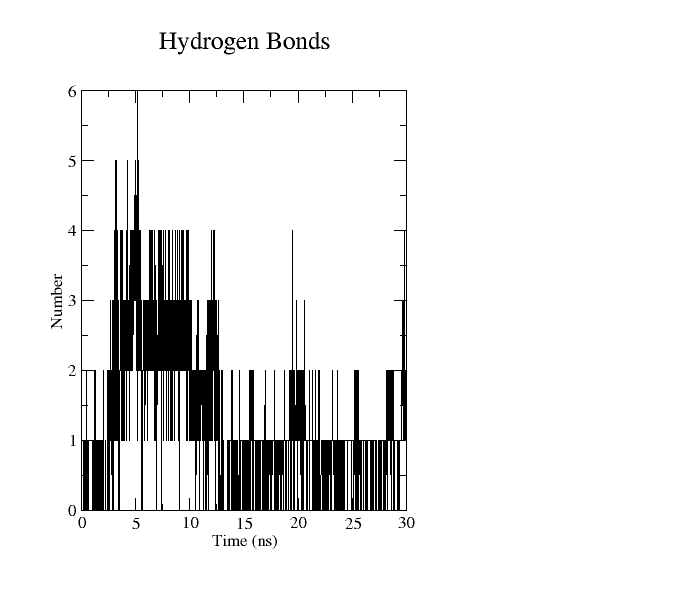 |
|  | **Remdesivir** | 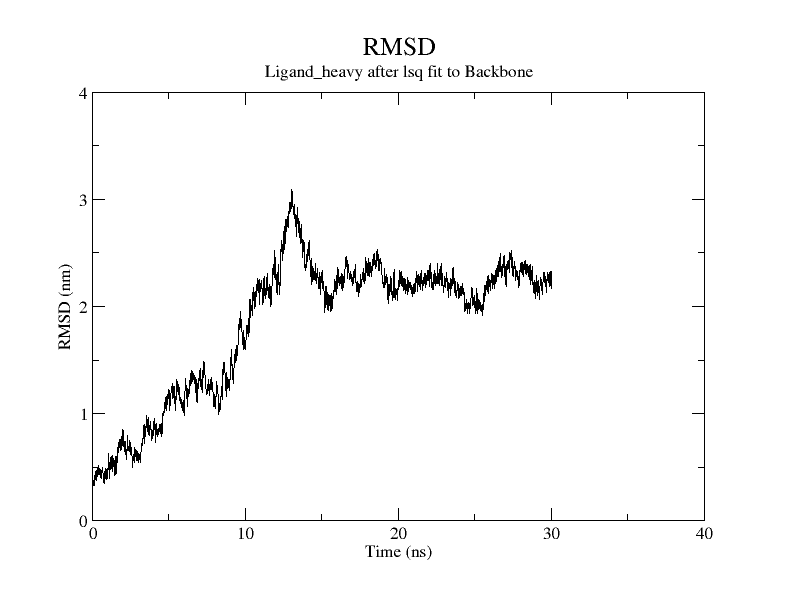 | 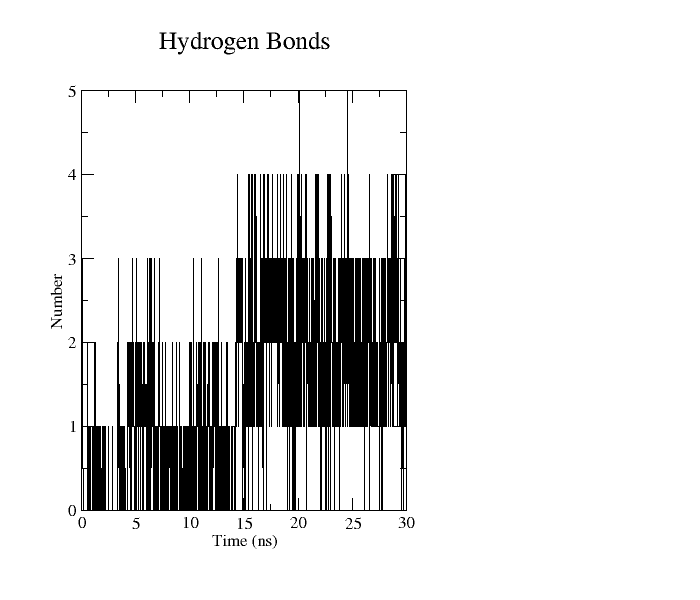 |
| **RdRp** | **Ivermectin** | 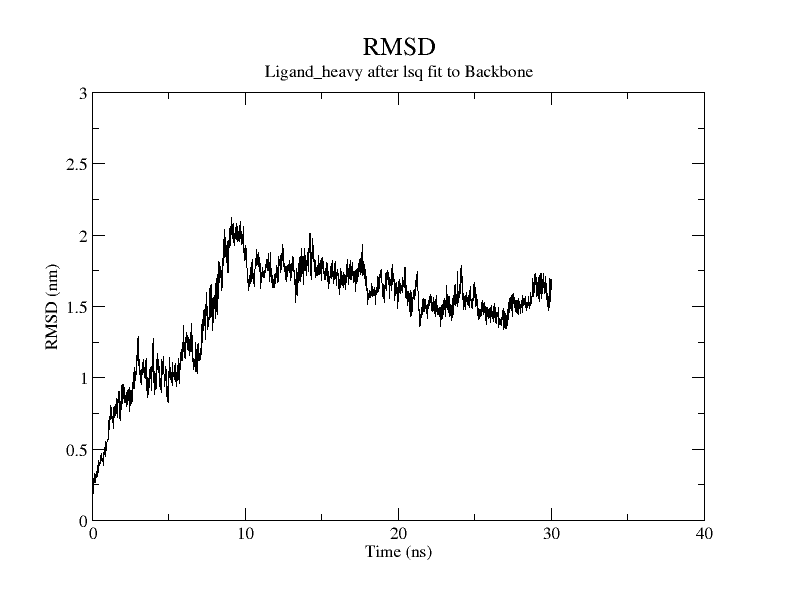 | 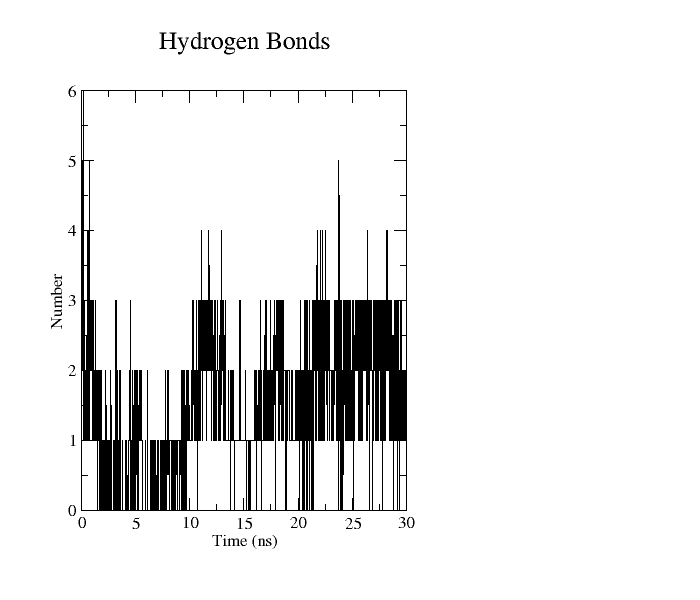 |
|  | **Remdesivir** | 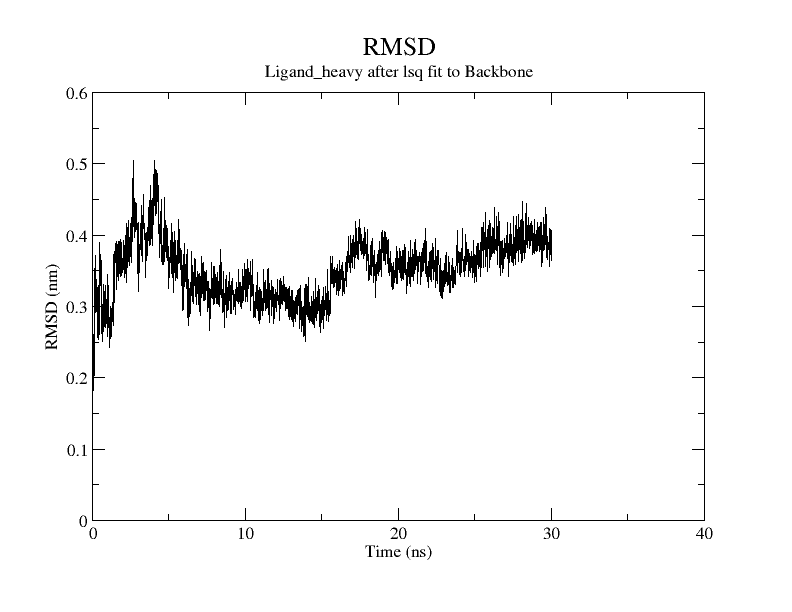 | 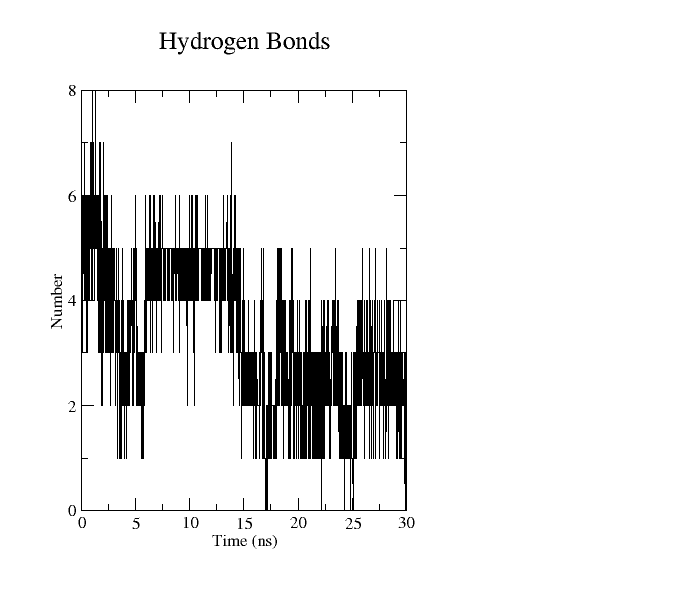 |
| **ExoN NSP14** | **Ivermectin** | 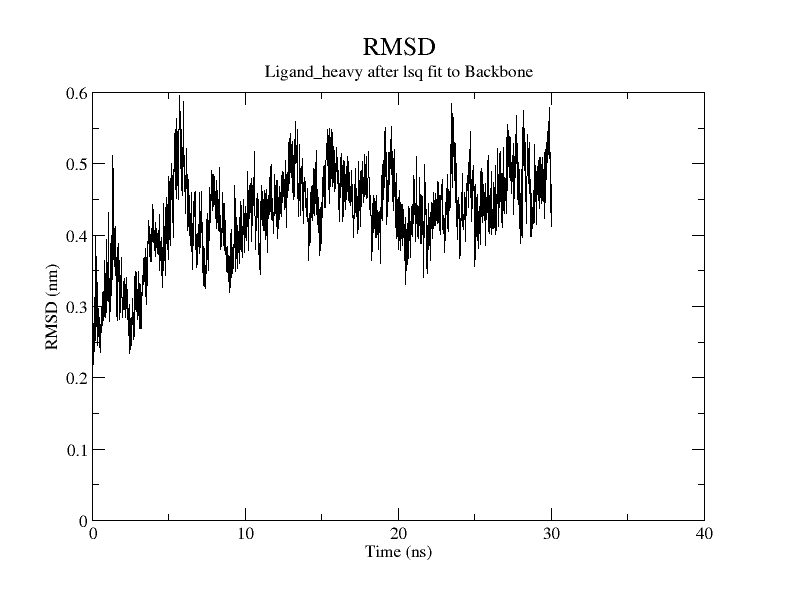 | 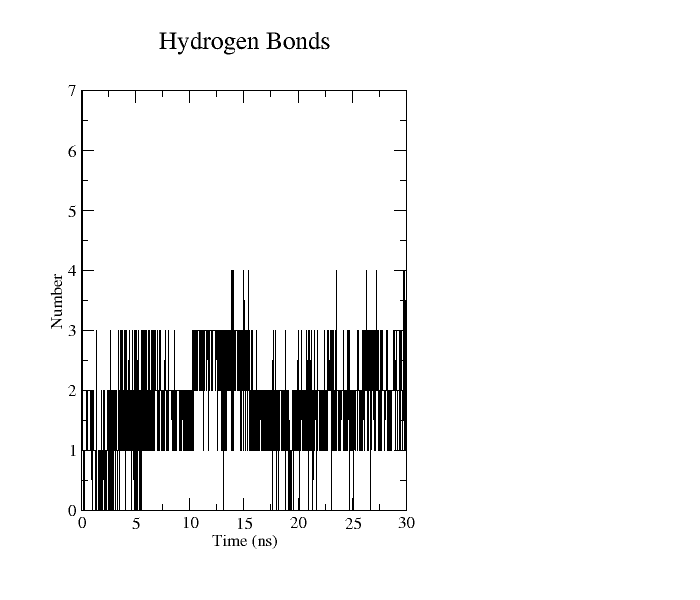 |
|  | **Remdesivir** | 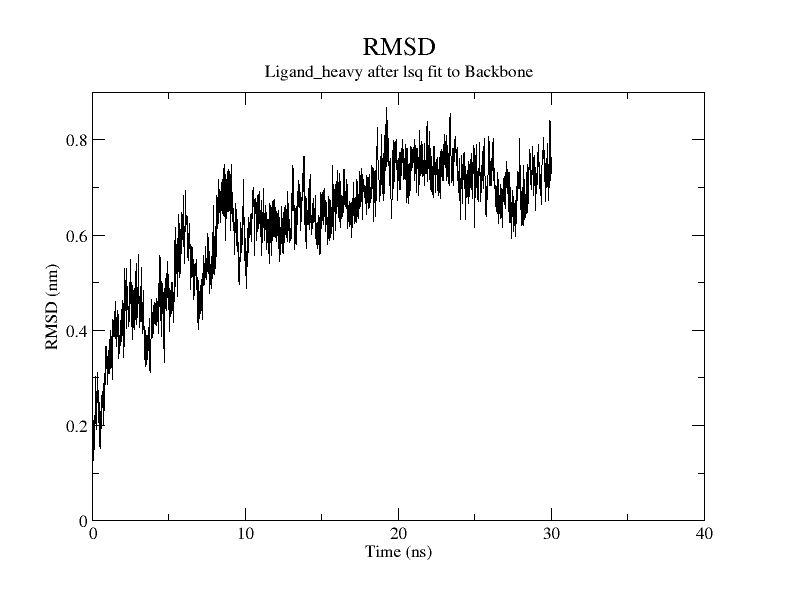 | 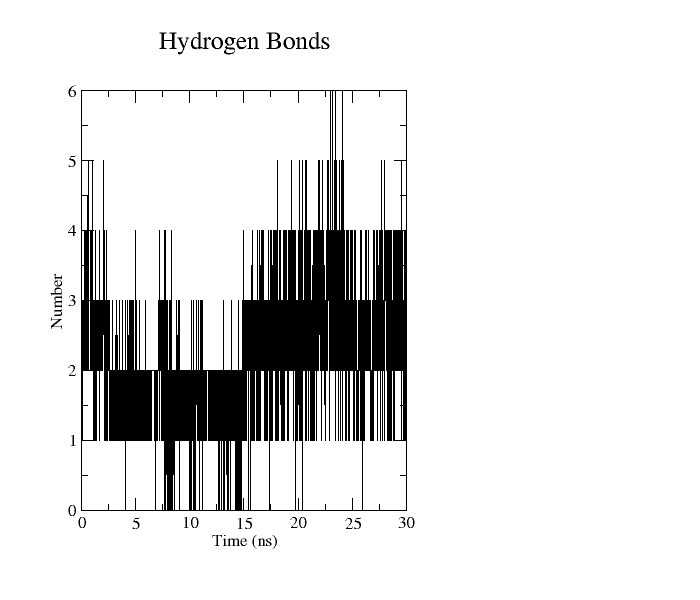 |
| **Main Protease (Mpro)** | **Lopinavir** | 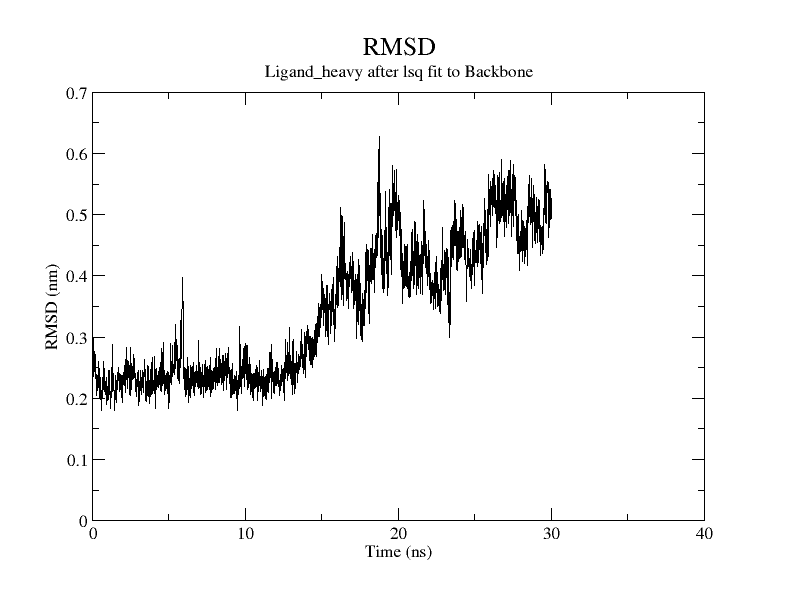 | 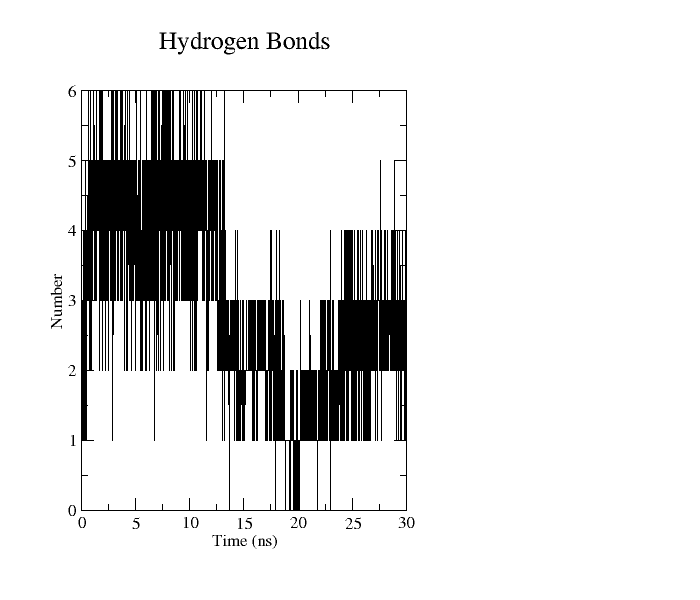 |
|  | **Ivermectin** | 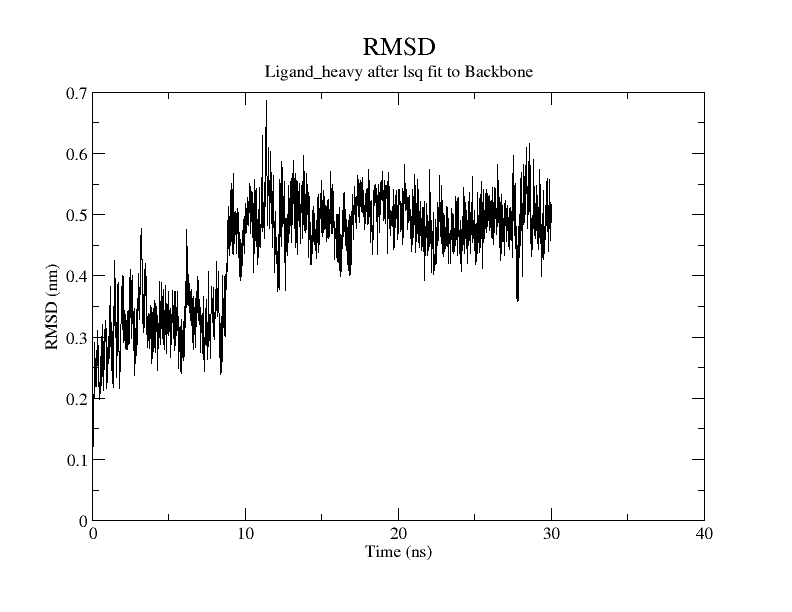 | 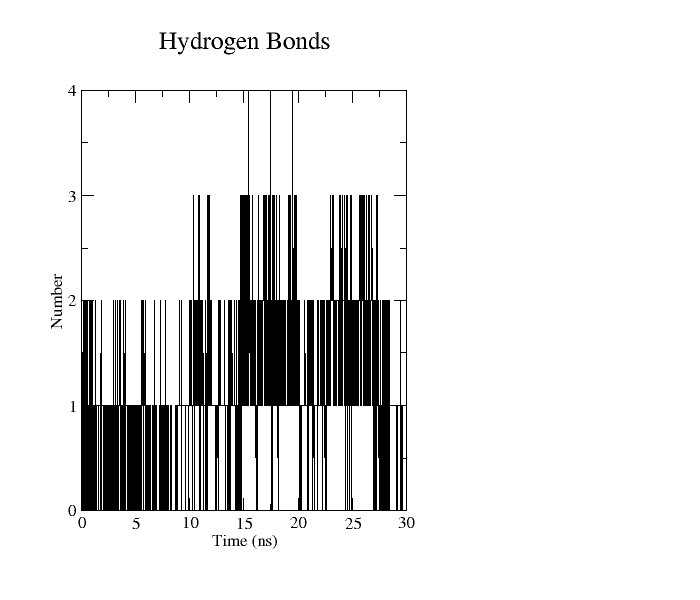 |
|  | **Remdesivir** | 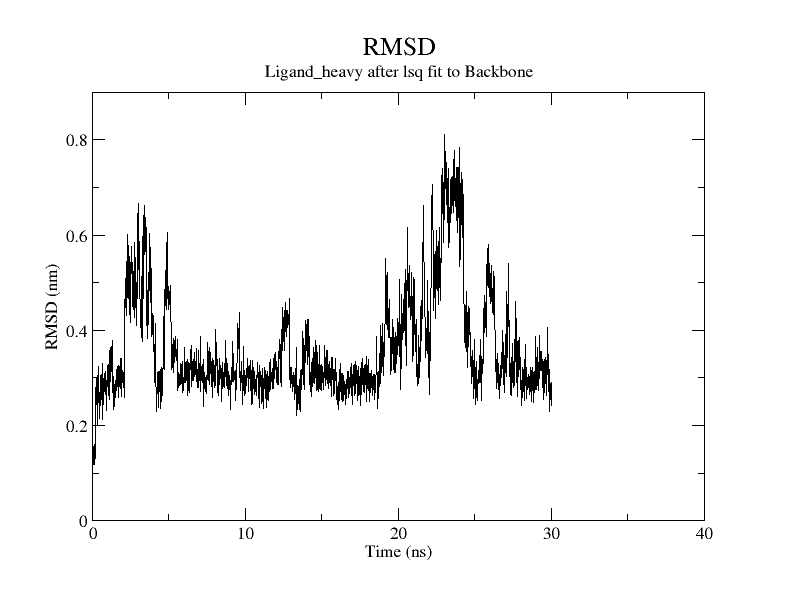 | 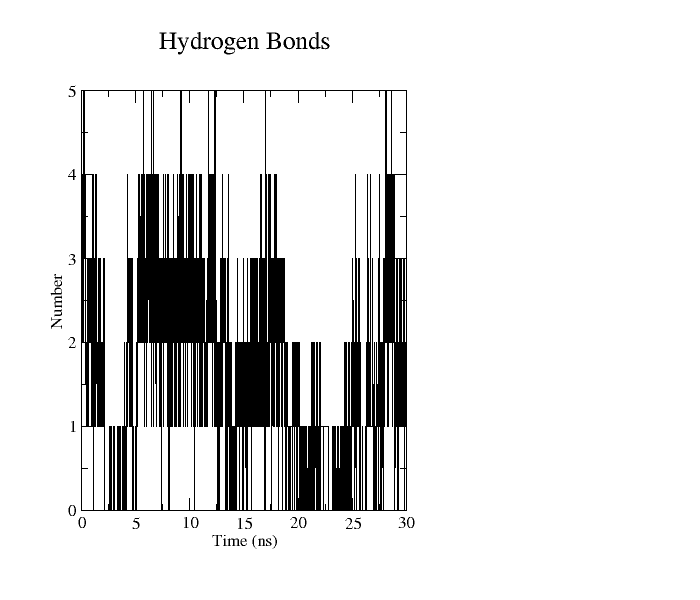 |
| **Papain-Like Protease (PLpro)** | **Lopinavir** | 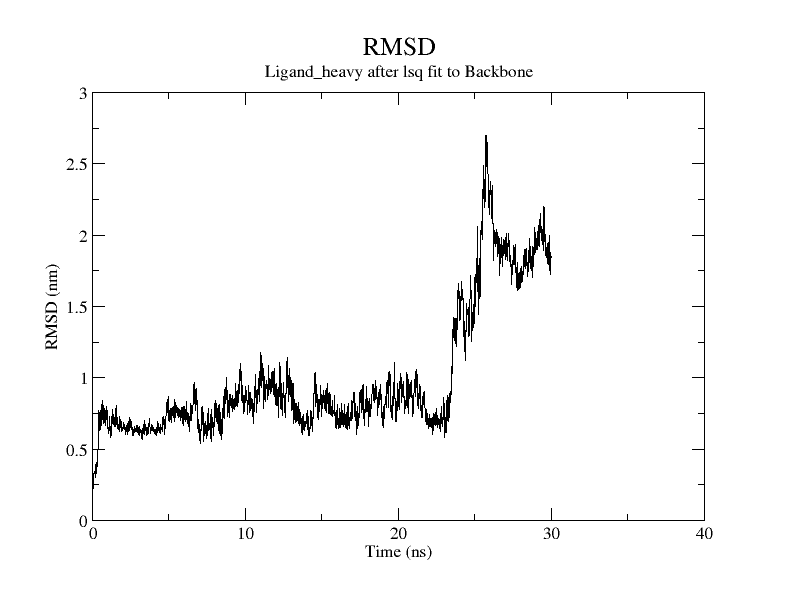 | 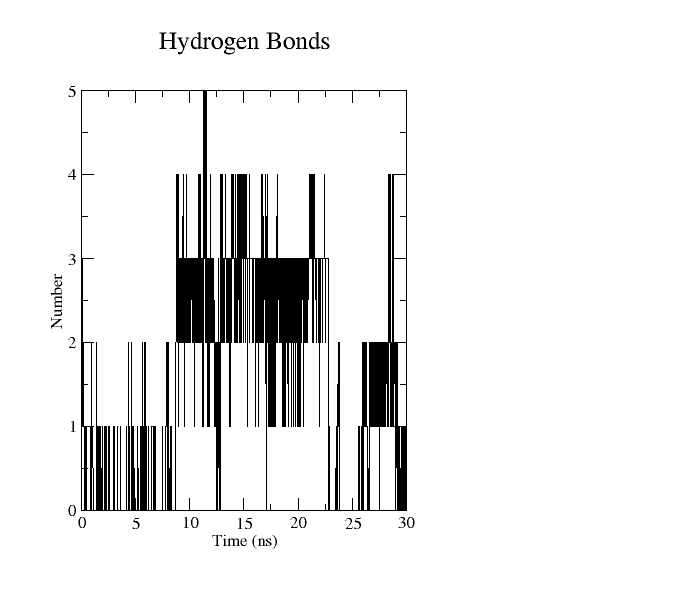 |
|  | **Ivermectin** | 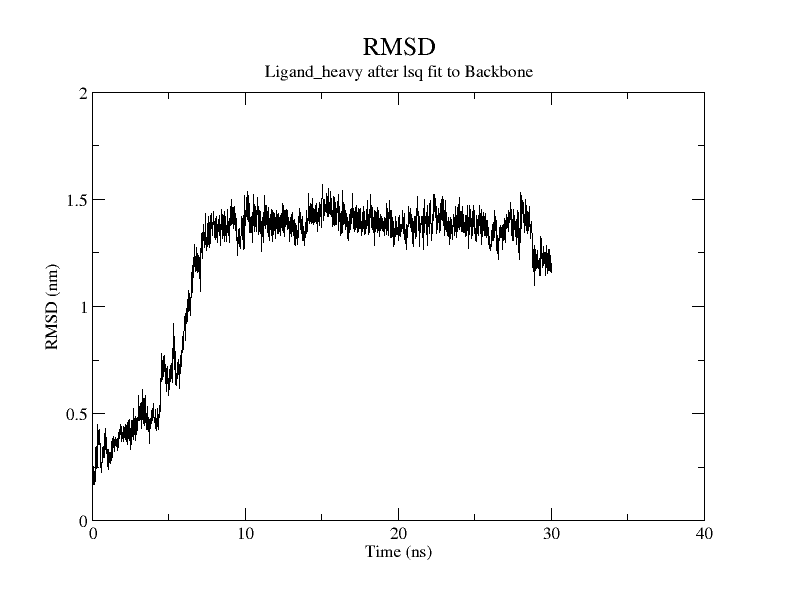 | 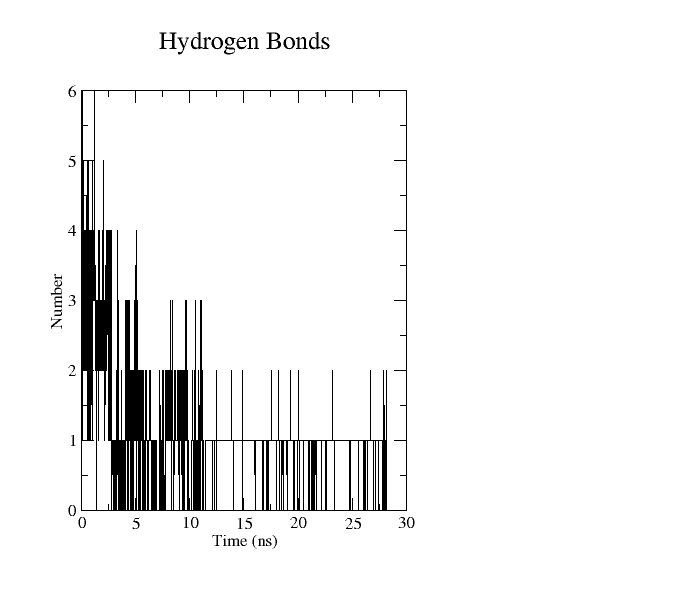 |
|  | **Remdesivir** | 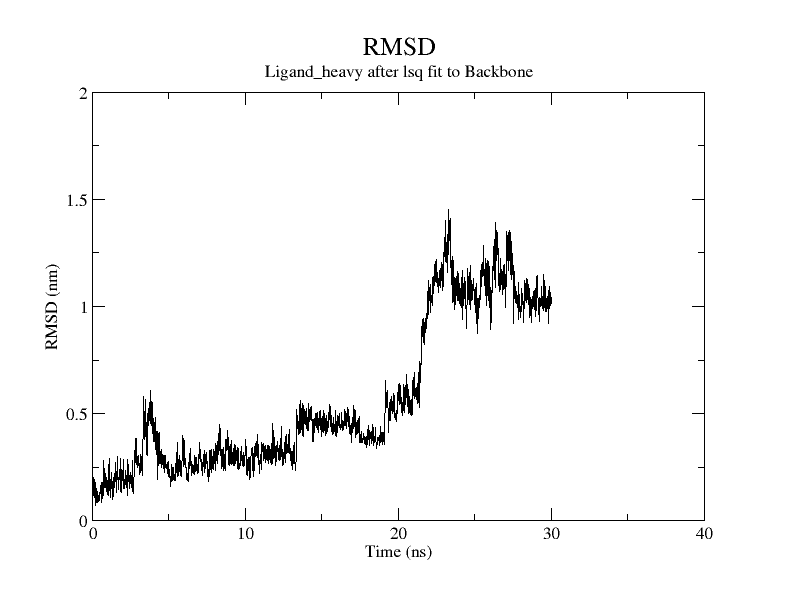 | 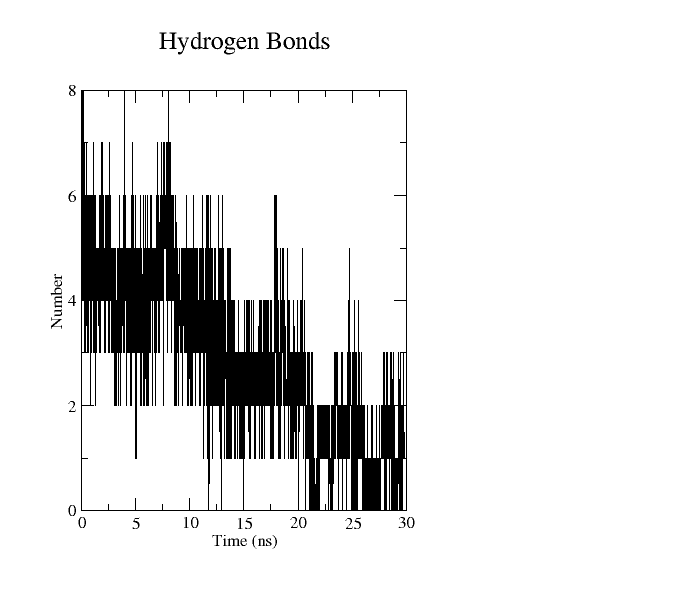 |
|  |  |  |  |
|  |  |  |  |
|  |  |  |  |
| **M-Protein** | **Ivermectin** | 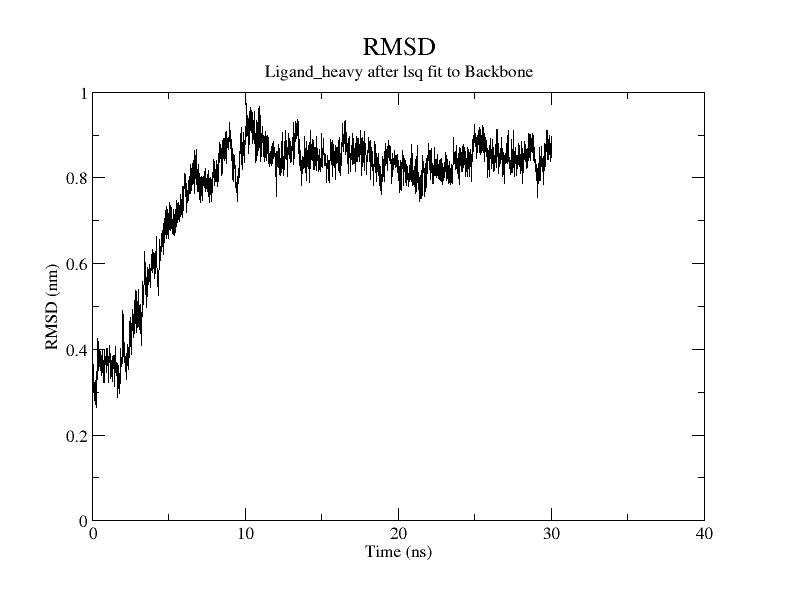 | 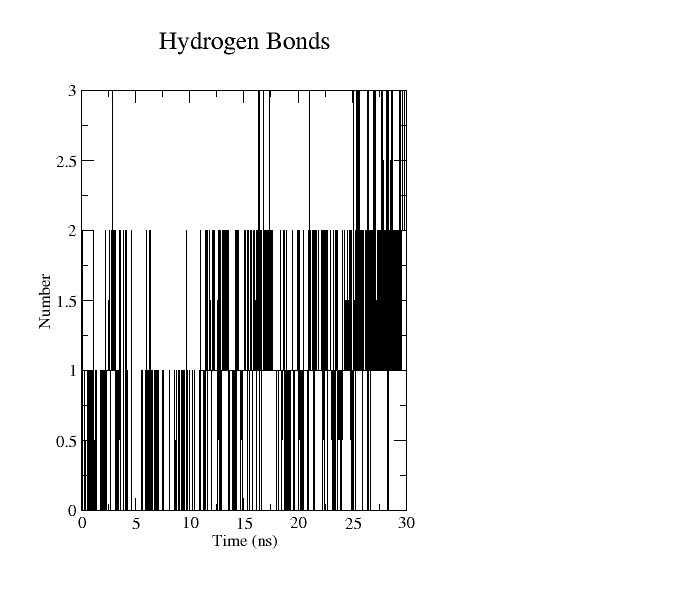 |
|  | **Remdesivir** | 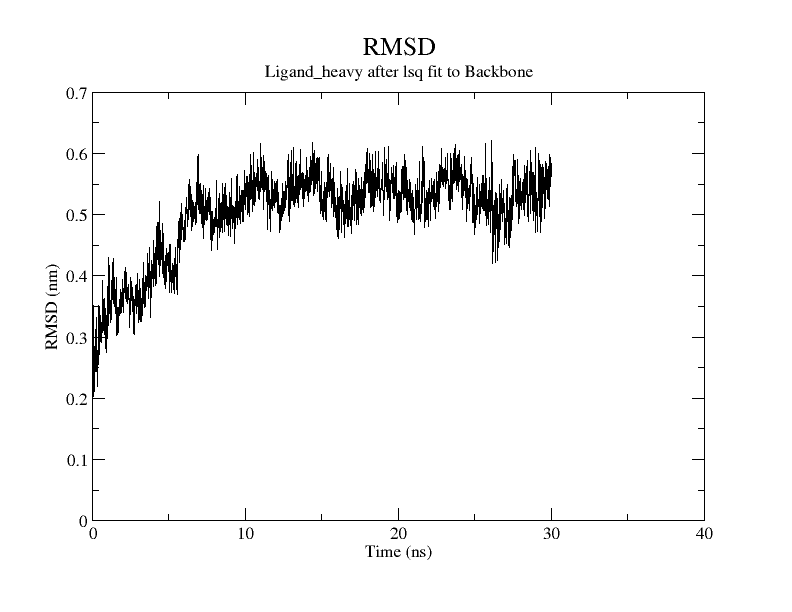 | 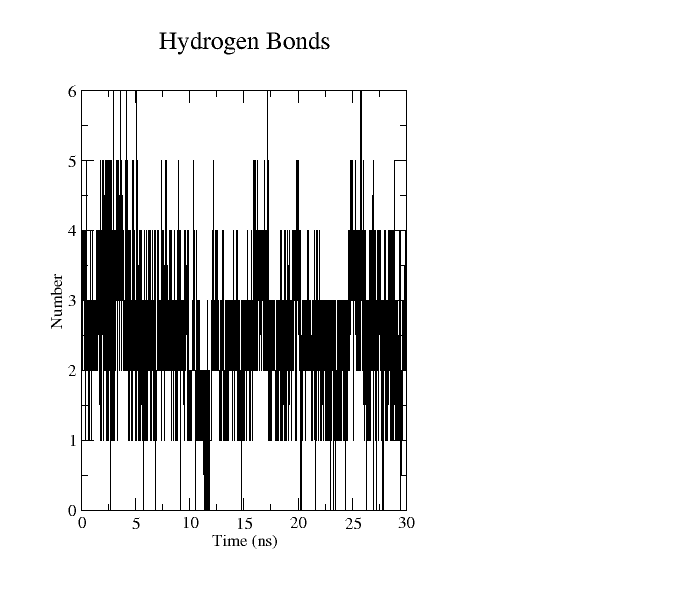 |
| **NP** | **Ivermectin** | 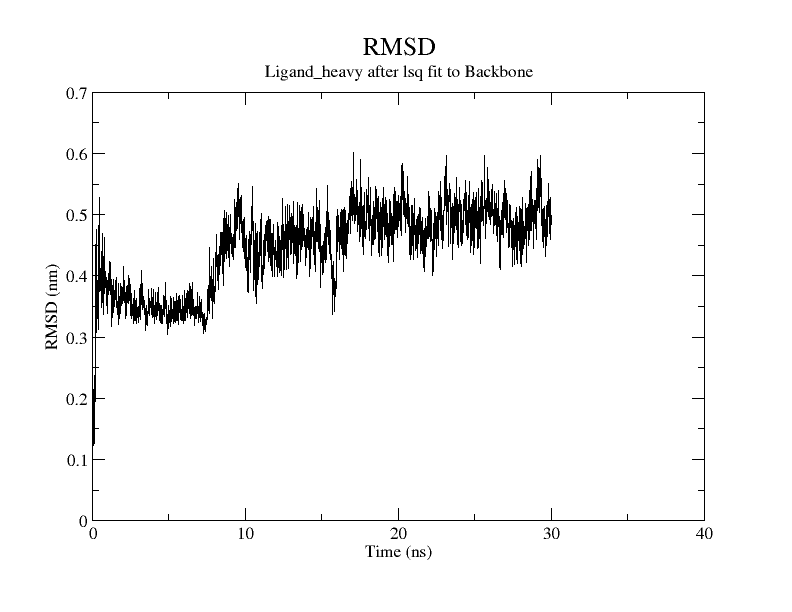 | 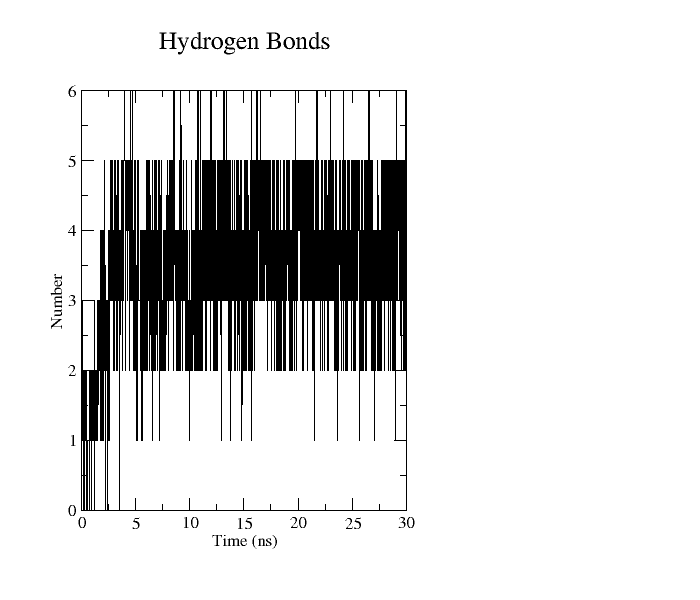 |
|  | **Remdesivir** | 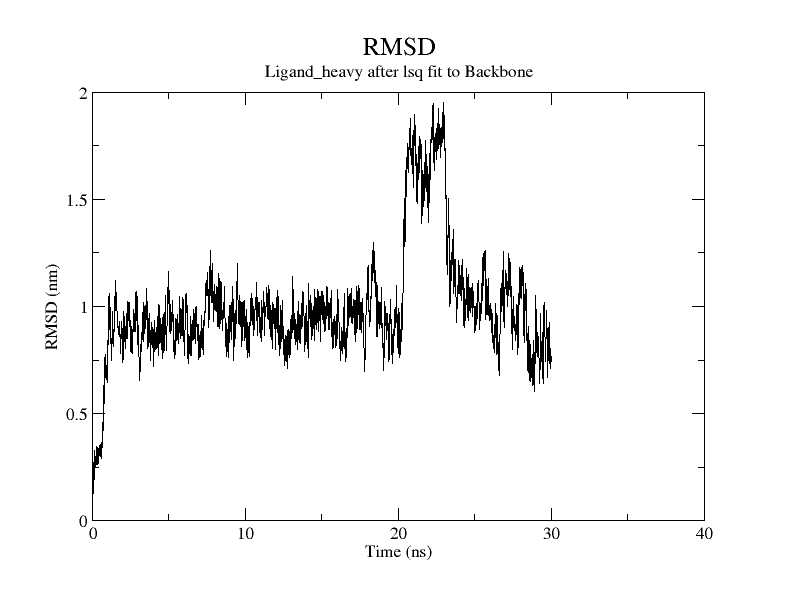 | 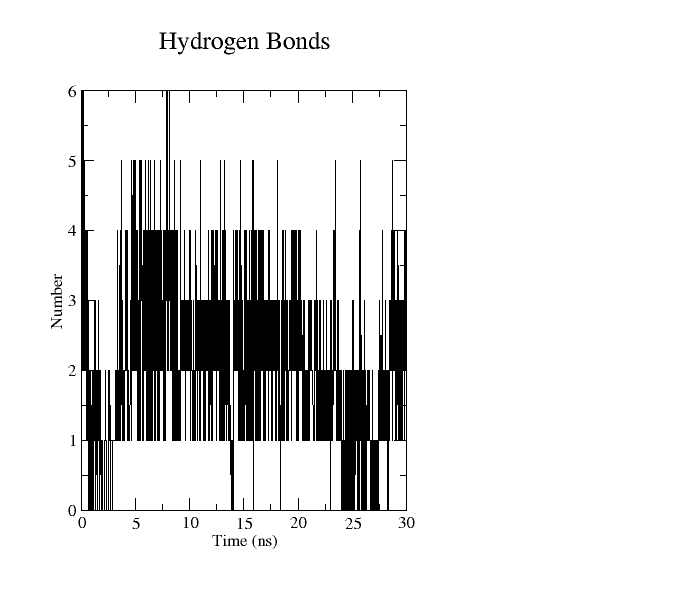 |
| **Human ACE-2** | **Ivermectin** | 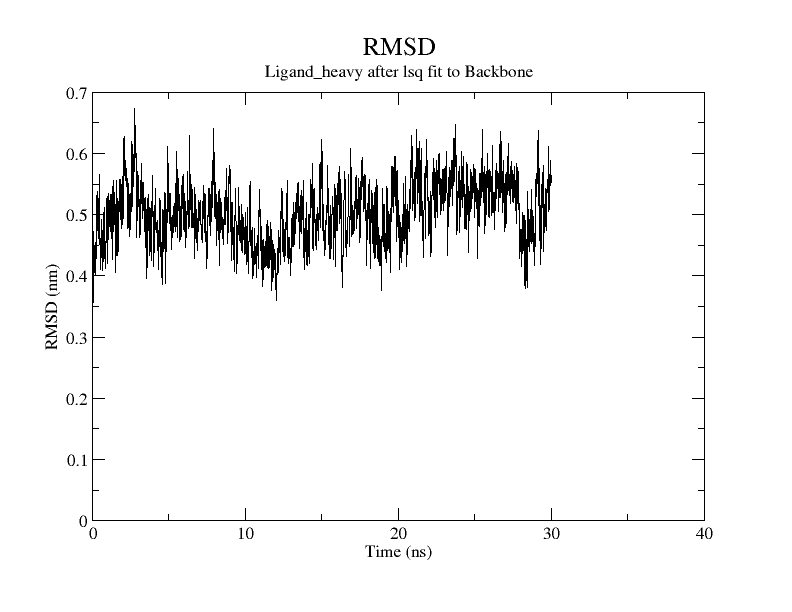 | 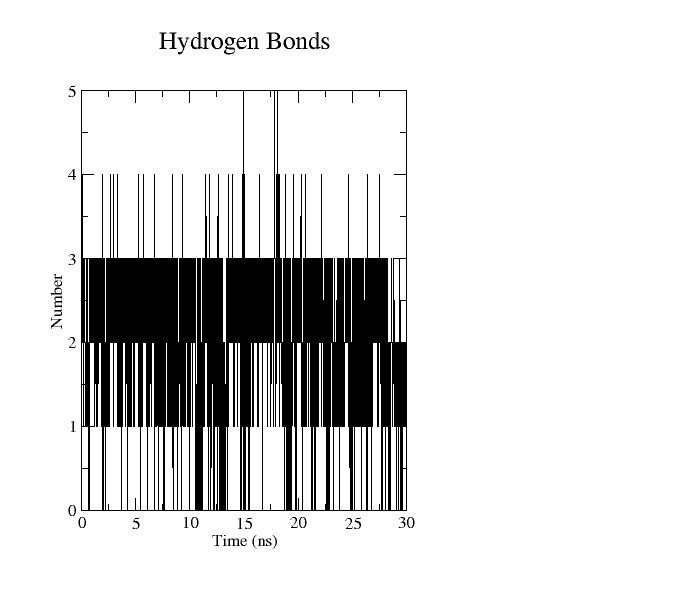 |
|  | **Remdesivir** | 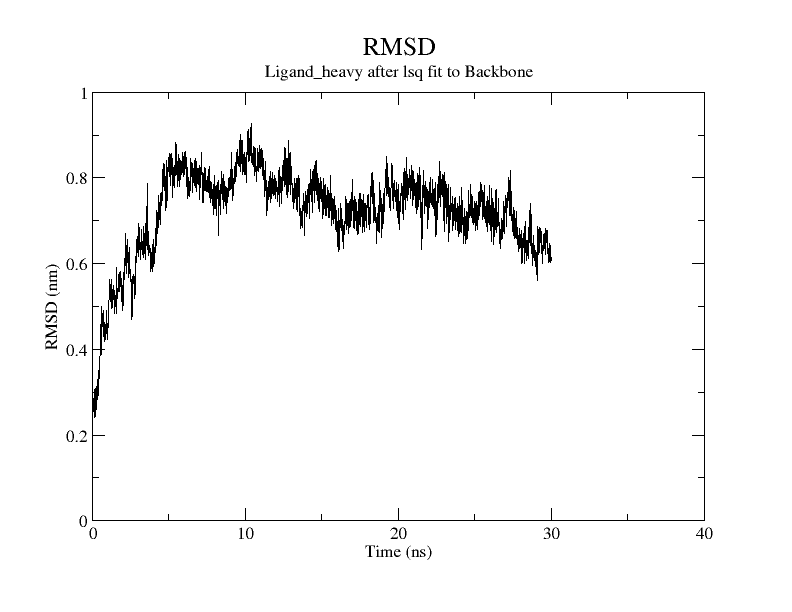 | 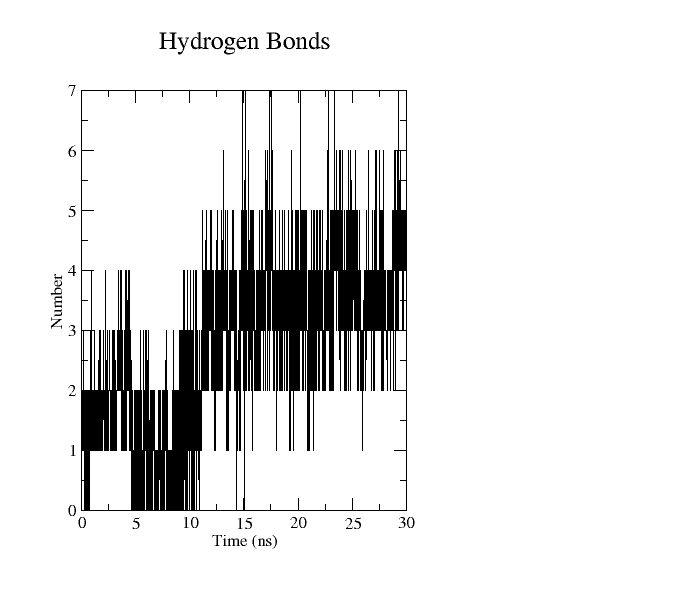 |
| **Human TMPRSS2** | **CAMOSTAT** | 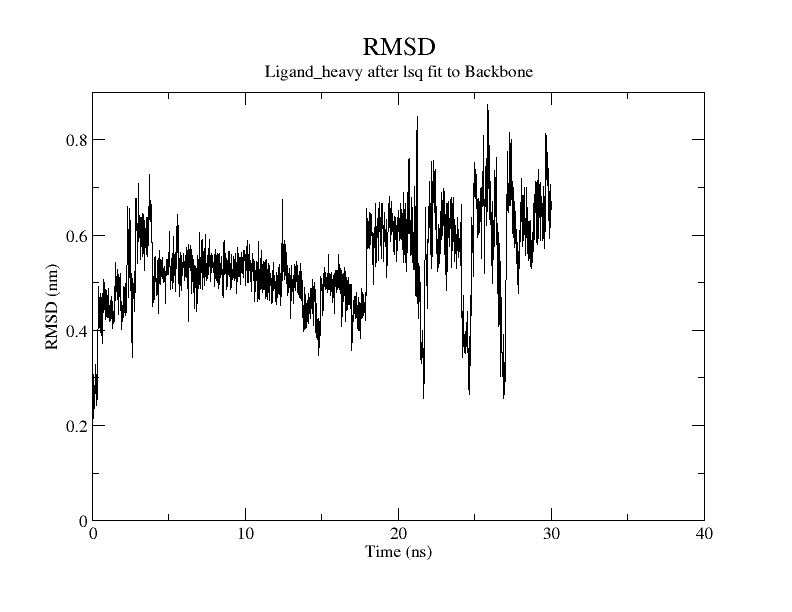 | 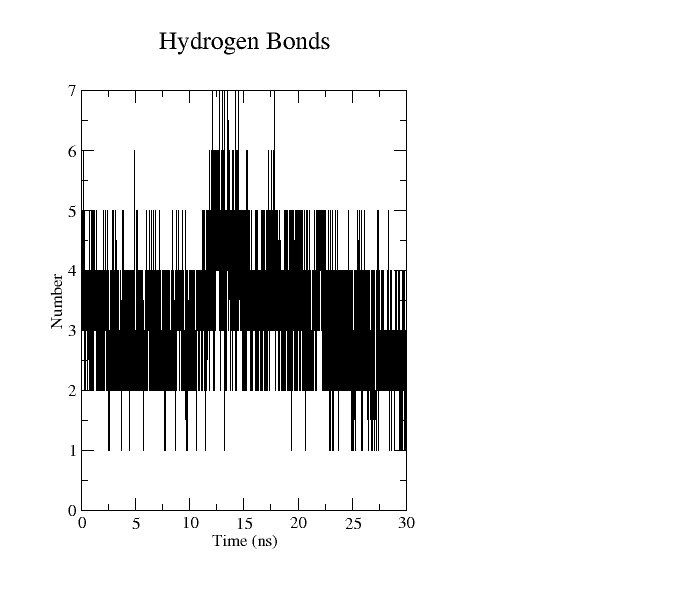 |
|  | **Ivermectin** | 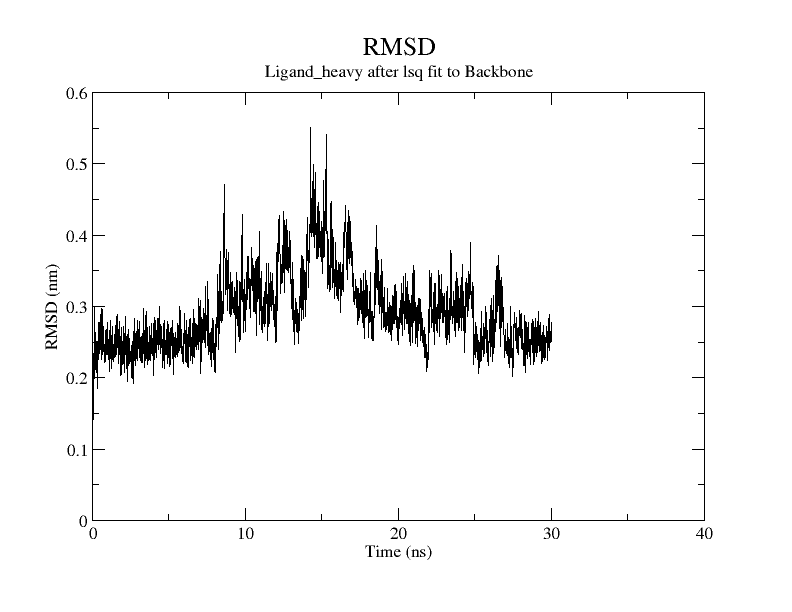 | 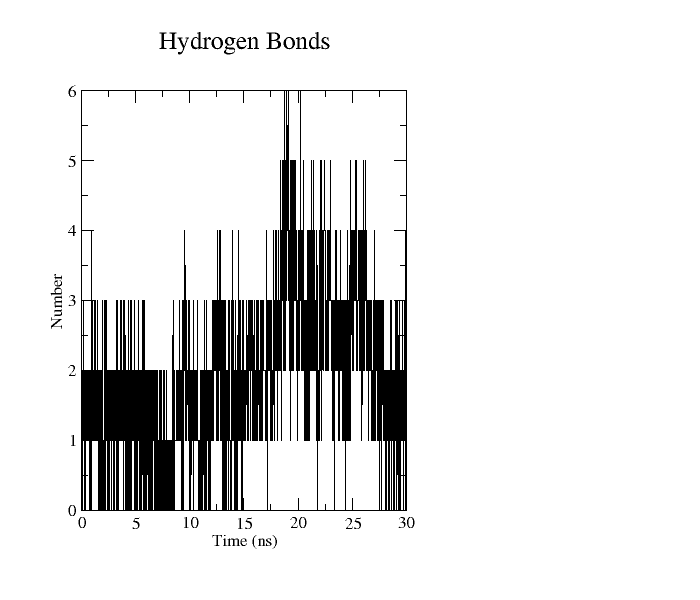 |
|  | **Remdesivir** | 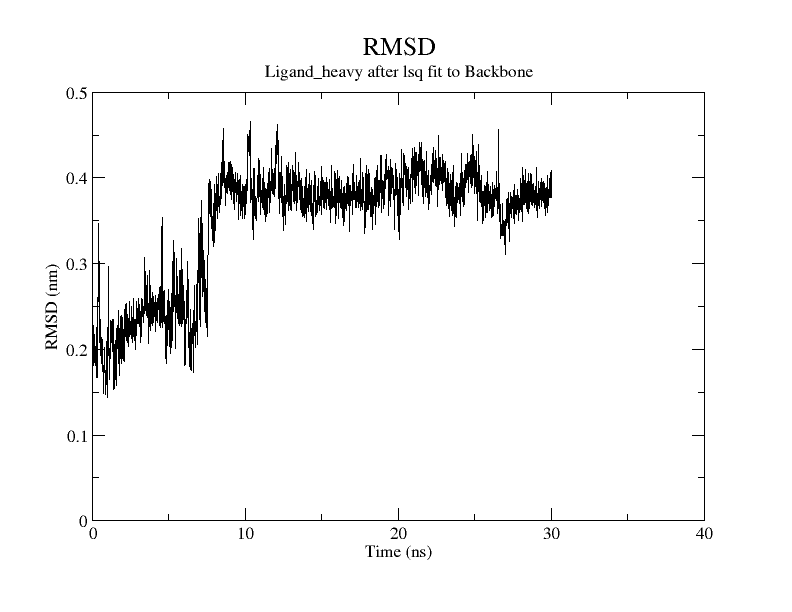 | 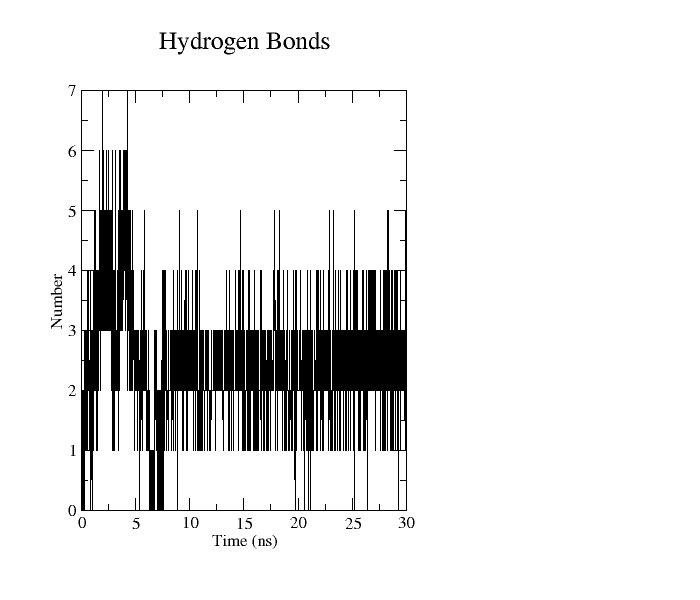 |
